# Understanding Drug Skin Permeation Enhancers Using Molecular Dynamics Simulations

**DOI:** 10.1021/acs.jcim.3c00625

**Published:** 2023-07-18

**Authors:** Christian Wennberg, Magnus Lundborg, Erik Lindahl, Lars Norlén

**Affiliations:** † Science for Life Laboratory, ERCO Pharma AB, 171 65 Solna, Sweden; ‡ Department of Biophysics and Biochemistry, Stockholm University, 106 91 Stockholm, Sweden; § Department of Applied Physics, Swedish e-Science Research Center, KTH Royal Institute of Technology, 106 91 Stockholm, Sweden; ∥ Department of Cell and Molecular Biology (CMB), Karolinska Institutet, 171 77 Solna, Sweden; ⊥ Dermatology Clinic. Karolinska University Hospital, 171 77 Solna, Sweden

## Abstract

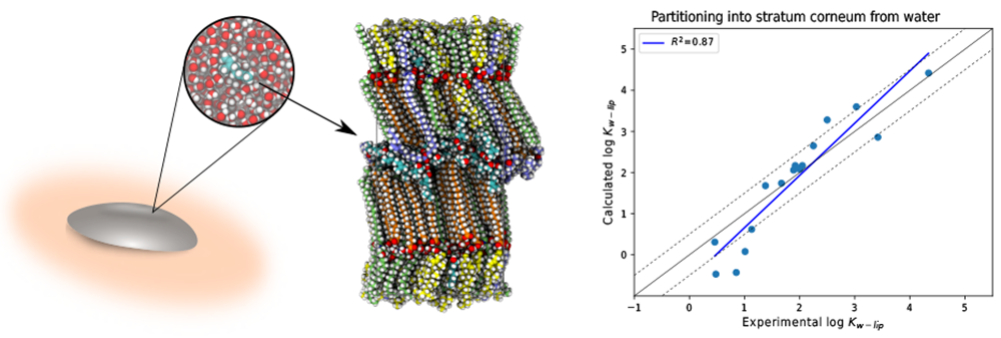

Our skin constitutes an effective permeability barrier that protects the body from exogenous substances but concomitantly severely limits the number of pharmaceutical drugs that can be delivered transdermally. In topical formulation design, chemical permeation enhancers (PEs) are used to increase drug skin permeability. *In vitro* skin permeability experiments can measure net effects of PEs on transdermal drug transport, but they cannot explain the molecular mechanisms of interactions between drugs, permeation enhancers, and skin structure, which limits the possibility to rationally design better new drug formulations. Here we investigate the effect of the PEs water, lauric acid, geraniol, stearic acid, thymol, ethanol, oleic acid, and eucalyptol on the transdermal transport of metronidazole, caffeine, and naproxen. We use atomistic molecular dynamics (MD) simulations in combination with developed molecular models to calculate the free energy difference between 11 PE-containing formulations and the skin’s barrier structure. We then utilize the results to calculate the final concentration of PEs in skin. We obtain an RMSE of 0.58 log units for calculated partition coefficients from water into the barrier structure. We then use the modified PE-containing barrier structure to calculate the PEs’ permeability enhancement ratios (ERs) on transdermal metronidazole, caffeine, and naproxen transport and compare with the results obtained from *in vitro* experiments. We show that MD simulations are able to reproduce rankings based on ERs. However, strict quantitative correlation with experimental data needs further refinement, which is complicated by significant deviations between different measurements. Finally, we propose a model for how to use calculations of the potential of mean force of drugs across the skin’s barrier structure in a topical formulation design.

## Introduction

Transdermal drug delivery enables continuous, noninvasive, and pain-free drug administration that, compared to oral or intravenous drug delivery, is associated with reduced side effects and increased patient compliance. Continuous drug administration for days to weeks, combined with the avoidance of first-pass metabolism in the gastrointestinal tract and liver, would give physicians greater control over the medication and dosage levels. The human skin is, however, an effective barrier to permeation, limiting the number of drugs presently available for transdermal delivery.

The skin’s main permeability barrier is located to a stacked lamellar lipid structure^1,2^ situated in the intercellular space of the superficialmost part of the skin, the stratum corneum.^3^ This lipid structure consists of a unique mixture of ceramides, cholesterol, and free fatty acids^4−6^ with a water content of approximately one to two molecules per lipid.^7,8^ A number of different models have been proposed for its detailed lipid packing.^8−15^

Today, the dominant means of assessing skin permeability is by *ex vivo*/*in vitro* testing in diffusion cells.^16^ These techniques often use excised skin, separating the donor and receptor chamber. To enhance transdermal drug transport, formulations containing one or more chemical permeation enhancers (PEs) are commonplace.^17^ However, the modes of action of PEs are often unknown, which limits their use in commercial applications. Various PE action mechanisms have been proposed (*cf.* ref (17)), including (i) altering the solubility of the drug in the formulation, (ii) modifying the partitioning of the drug into the skin’s barrier structure, (iii) changing the thermodynamic activity of the drug, (iv) promoting transport by “dragging” the drug across skin, and (v) perturbing the lipid organization of the skin’s barrier structure. In addition, many PEs have complex and nonlinear concentration-dependent permeability-enhancing effects. In order to understand these effects, the partitioning of various formulation excipients into the stratum corneum has been investigated.^18−20^ Furthermore, the effect of PEs on the structural properties of the stratum corneum has been studied using an array of different experimental methods, including differential scanning calorimetry, infrared spectroscopy, small-angle X-ray diffraction, and solid-state NMR spectroscopy.^21−23^

*In silico* modeling can offer additional insight into the absorption of chemicals into and permeation of chemicals across skin. A detailed understanding of how PEs interact with the skin’s barrier structure could enable a tailored design of topical drug delivery vehicles, ensuring that they modify the barrier in regions that present the highest permeation resistance to a specific drug. Quantitative structure–permeability relationship (QSPR)^24−28^ methods are often used to predict permeability coefficients for molecules permeating the skin. QSPR models are good at predicting drug permeability coefficients, with reported mean square errors as low as 0.48 cm^2^ h^–2^ (log units) and *R*^2^ = 0.7.^25,28^ However, due to the wide range of molecular mechanisms for the actual permeation process, QSPR methods are limited with respect to faithfully modeling the permeability-enhancing effects of PEs, as the methods will need to have been trained on PEs that have both similar structures and similar permeation-enhancing molecular effects. The general prediction performance of QSPR methods is thus reduced for new categories of PEs, and the methods are unable to explain the mechanism of action of PEs.

Molecular dynamics (MD) simulations are not as domain-limited as QSPR methods. In addition, they enable the incorporation of PEs into the skin’s barrier structure at their most probable concentrations, locations, and orientations. The atomistic resolution of MD simulations can yield an understanding of how PEs and other formulation excipients interact with the barrier in order to modify the permeation of various drugs as well as potential direct interactions with the barrier lipids. It should be noted that the atomistic models are relatively simplistic, and detailed knowledge about the exact lipid organization in the human skin barrier is still evolving. Furthermore, the currently used models do not contain any representation of corneocytes or the cornified cell envelope. However, the ability of MD simulations to address how various formulation excipients influence skin permeability makes them an attractive alternative or complement to QSPR methods with respect to transdermal drug delivery design. MD simulation modeling of skin lipid bilayers has been performed by several groups,^29−35^ often based on hairpin bilayers consisting of ceramides, cholesterol, and free fatty acids. More complex structures incorporating ceramide EOS have also been employed,^36,37^ yielding better agreement with experimentally measured permeability values.

Recently, we have presented an improved simulation protocol for transdermal drug transport modeling, which enables improved precision through better sampling of the free energy landscape as well as a better calculation of the local diffusion coefficient of drugs permeating the skin’s barrier structure.^38^ Unfortunately, good reference *in vitro* skin permeation data are scarce, largely due to variability of reported values between laboratories (*e.g.*, roughly 1 order of magnitude for testosterone^39,40^) and/or to differences in experimental setups (*e.g.*, in donor concentration, temperature, skin thickness, donor/receptor fluids) which impede direct comparison. In this work, we selected two recent studies for testing our modeling approach. These studies were chosen to avoid comparing permeability data on the same permeant from different laboratories and to ensure that the reported permeability enhancement was obtained using an identical experimental setup. First we compare our results with those of Pham et al.,^23^ who reported the effect of saturated solutions of single PEs on skin permeation of metronidazole. Then we focus on selected data from the work of Abd et al.,^41^ which involves the permeability-enhancing effect on caffeine and naproxen of three formulations based on ethanol and water. This was assumed to pose a greater challenge due to the differing ionization of naproxen in the different formulations but enabled the possibility to compare the effect of similar formulations on different permeants. We also decided to not include comparisons with sodium lauryl sulfate due to expected difficulties of modeling charged molecules. Finally, we outline our basic general approach with respect to MD skin permeability modeling and address the challenges ahead.

For more detailed information about the simulation methods used and the atomistic skin barrier model employed, the reader is referred to previous publications.^8,38,42^

## Methods

The atomistic model of the human skin’s barrier structure presented by Lundborg et al.,^8^ and originally called 33/33/33/75/5/0.3,^a^ was used as the starting structure for the simulations. The starting model had been equilibrated for approximately 270 ns, 250 ns of which was without restraints.^8^

All MD simulations in this study were performed using GROMACS 2022,^43−45^ using the accelerated weight histogram (AWH) method for alchemical free energy calculations.^42,46,47^ A source code modification enabled symmetrizing AWH sampling along a spatial (pulling) reaction coordinate dimension and also changed the number of AWH blocks for autocorrelation analysis from 64 to 128. These changes are available from the GROMACS gitlab repository^48^ and are expected to be included in future releases of the code. Symmetrizing effectively improves the convergence of the simulations. We have previously not experienced any significant difference in the results when comparing PMFs calculated with and without symmetrization (see Figure S1 in ref (49)).

Van der Waals interactions had a cutoff of 1.2 nm with a smooth force switch from 1.0 to 1.2 nm. The simulations were run without a dispersion correction for the energy and pressure. Coulomb interactions were calculated using PME^50^ with a radius of 1.2 nm. Bonds to hydrogen atoms were constrained using the P-LINCS algorithm.^51,52^ TIP3P^53^ parameters were used for water molecules. For the lipid molecules, the CHARMM36 lipid force field^54,55^ was used. Ceramide parameters were modified to more accurately reproduce the ceramide NP crystal structure^56^ as described in ref (8). In order to allow a 3 fs integration time step, hydrogen atoms were made 3 times heavier by repartitioning the corresponding mass from their bound heavy atoms.^57,58^ The temperature was set to 305.15 K by using a stochastic dynamics integrator (also referred to as a velocity Langevin dynamics integrator) with a time step of 3 fs and a time constant (τ) of 2 ps (corresponding to a friction constant of 0.5 ps^–1^). The pressure was set to 1 atm and controlled using a stochastic cell rescaling barostat^59^ with a time constant of 1.0 ps and a compressibility of 4.5 × 10^–5^ bar^–1^. In all simulations containing the skin barrier system, the pressure coupling was semi-isotropic with no compressibility in the *Z* dimension to maintain the lateral spacing derived from the original CEMOVIS data.

Along the alchemical free energy dimension, 21 equidistantly distributed λ states were used for decoupling both van der Waals and Coulomb interactions simultaneously. There were 100 steps between each Monte Carlo coordinate sampling along the alchemical free energy λ value and spatial position across the lipid structure. There were 10 samples per update of the *f*_λ_ bias. The estimated initial error was set to 10 kJ mol^–1^. Soft-core transformations^60^ with α = 0.5 and σ = 0.3 nm were applied to both the van der Waals and Coulomb interactions of the solute.

Topologies (*i.e.*, inter- and intramolecular interaction parameters) for all permeants and formulation components except water were generated using STaGE,^61^ which in turn uses Open Babel^62^ and MATCH^63^ to generate GROMACS topologies compatible with the CGenFF^64^ CHARMM force field.

All images representing molecules were prepared using Tachyon^65^ in VMD.^66^

### Solvation Free Energy Calculations

The solvation free energy was calculated starting with a box large enough to solvate the solute (system sizes for all formulations simulated in this work are shown in Table S5). For each solvation free energy calculation, the solute was inserted into the system at a random position with its interactions to the surroundings turned off, as if in vacuum. The temperature was set to 305.15 K. AWH was used to sample the alchemical free energy λ states^42^ to calculate the solvation free energy. The initial AWH histogram size, which determines the initial update size of the free energy, was set indirectly by specifying an estimate of the diffusion constant along the alchemical free energy λ axis in combination with a rough estimate of the initial error. An input diffusion constant of 1 × 10^–3^ ps^–1^ was used for the solvation free energy calculations, which means that it is estimated to take approximately 1 ns to cross the alchemical dimension for one AWH walker. Twenty-four communicating AWH walkers were run in parallel, with the requirement that only simulations that covered the whole alchemical reaction coordinate counted toward the covering check in the initial AWH stage. The simulations were 60 ns long per walker for a total simulation time of 1440 ns per solute.

### Permeability Coefficient Calculations

The permeability coefficient, *K*_P_, and the permeation resistance, *R*, were calculated from the obtained potentials of mean force (PMFs) across the skin’s barrier structure as follows:^67^
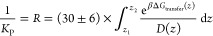
1where Δ*G*_transfer_ is the free energy relative to the solvation free energy, β is the thermodynamic β, and *D* is the local diffusion coefficient across the skin’s barrier structure (d*z*). The PMFs were shifted so that the PMF minimum was never below 0, and following the discussion in ref (38), the factor 30 ± 6 represents an estimate of the number of lipid bilayers present in the stratum corneum based on observations from cryo-EM images of human skin.^14,68^ Since the data presented herein mostly focus on relative differences between simulated and *in vitro* enhancement ratios, the number of assumed bilayers does not influence the final conclusions.

The AWH simulation estimates a friction metric tensor *g*(*z*),^69^ which can be used to derive the diffusion coefficient across the barrier structure, *D*(*z*) = *g*^–1^(*z*). To reduce the noise of the local diffusion coefficient curves, a 0.2 nm wide rolling median filter was applied. When symmetrizing the sampling along the spatial dimension by using the absolute coordinate values and accounting for the AWH bias across the sampling boundaries, there are usually artificial spikes at the edges of the PMFs. These were removed by setting the two lowest points (0.005 and 0.015 nm) and two highest points (5.200 and 5.210 nm) in the PMF to the values of their neighbors (0.025 and 5.19 nm, respectively). These minor adjustments were made merely to avoid strange-looking features in the curves and are far smaller than any statistically significant effects on the calculated permeability coefficients.

At the start of each AWH walker simulation, the permeating molecule was inserted in the lipid barrier structure at a random position with all interactions to its environment turned off. The free energy profile through the skin’s barrier structure was calculated with a two-dimensional AWH setup using a harmonic potential to steer the permeant along the direction normal to the barrier plane (also referred to as the *Z* dimension) and an alchemical free energy reaction coordinate.^42^ This allows sampling of the free energy along the permeation direction and also the relative insertion free energy of the permeant from vacuum. In turn, this enables a direct calibration to the solvation free energy since the vacuum state is the same in both cases. The AWH input diffusion constant was set to 3 × 10^–5^ nm^2^ ps^–1^ for the spatial pulling dimension and 5 × 10^–5^ ps^–1^ along the alchemical free energy dimension. The input diffusion constant affects only the AWH histogram size but not the computed diffusion coefficient, which is obtained from the AWH friction metric during data analysis. The AWH force constant along the spatial *Z* dimension (normal to the lamellar stack), steering the permeant relative to the ceramide fatty acid chains using a harmonic pull potential, was set to 25 000 kJ mol^–1^ nm^–2^. The force constant also determines the resolution along the reaction coordinate dimension. For each permeant, three sets of simulations were run with heavy hydrogen atoms (see above) and a 3 fs integration time step. These were run using 24 communicating walkers, each running for 450 ns. The combined PMF was derived from the average of the independent PMFs from the three sets of simulations.

Along the alchemical free energy dimension, it is the end states, *i.e.*, the fully interacting and fully decoupled states, that are of highest interest, as the difference in free energy between them corresponds to the probability of transferring the permeant from vacuum into the skin’s barrier structure. Therefore, the target distribution used in these simulations puts more weight on the end states, especially the state with interactions fully turned on. The target distribution along the alchemical free energy dimension that was used is the same as shown in the supplement of ref (38). Along the spatial pulling dimension, the target distribution was uniform.

### Skin Partition

From the solvation free energy and the PMF we calculate the partition coefficient of the solute into the skin barrier structure as follows:
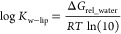
2where Δ*G*_rel_water_ is the Boltzmann-weighted free energy in the lipid system relative to water, *R* is the gas constant, and *T* the temperature.

### Insertion of Permeation Enhancers

**Figure 1:**
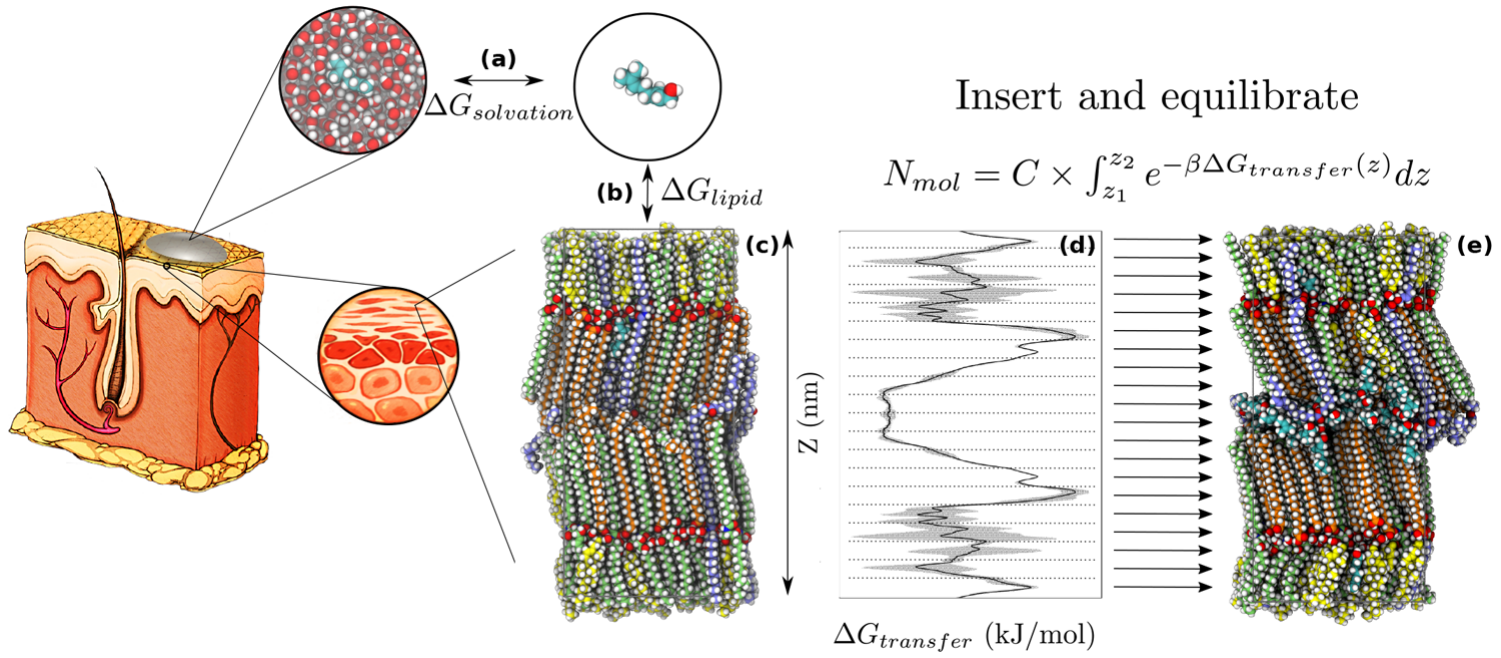
**Insertion of chemical permeation enhancers.** Procedure for the insertion of a permeation enhancer (PE) into the skin’s barrier structure, using the geraniol system in Table 1 as an example. (a) The solvation free energy, Δ*G*_solvation_, of the PE in the applied formulation is calculated relative to a vacuum. (b, c) The free energy of insertion, Δ*G*_lipid_ (with vacuum as the reference state), as well as the pulling free energy through the skin’s barrier structure are calculated using the two-dimensional accelerated weight histogram method. (d) By combining the results obtained in (a–c), the free energy difference between the formulation and the different parts of the skin’s barrier structure, Δ*G*_transfer_, can be calculated. This free energy is then used to calculate the number of molecules to insert along the *Z* dimension of the system according to eq 3. (e) The final system is equilibrated and can then be used for permeability calculations of selected permeants. Carbon atoms are colored as follows: in nonacyl ceramides, green; in acyl ceramide EOS, light blue; in free fatty acids, orange; in cholesterol, yellow; in geraniol, teal. The rest of the atoms are colored according to atom type (oxygen, red; nitrogen, blue; hydrogen, white). The illustrated skin patch in the image is adopted with permission from refs (38) (CC BY 4.0) and (70) (copyright 2016 C. Wennberg).

Due to the limited knowledge about the structure of the surface at the point where the applied formulations contact the skin as well as the associated large computational cost, it is unfeasible to perform a direct simulation of the partitioning of PEs into the skin’s barrier structure. Instead, we utilize the PMFs obtained for each PE through the reference bilayer structure (without any added excipients) to calculate the total number of PEs to insert from the formulation into the skin system, *N*_mol_, as follows:
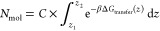
3where Δ*G*_transfer_ is the free energy relative to the solvation free energy in the formulation, β is the thermodynamic β, and the constant *C* is the number of PEs in the formulation corresponding to the same volume as the simulated skin bilayer. In order to avoid inserting the PEs in regions where they have a low probability of partitioning into, we divided the system into 20 slices along the *Z* direction (normal to the bilayer) and performed the calculation in eq 3 within each slice. The PEs were then inserted at random positions in the lateral *XY* plane within their corresponding slice with their interactions with their surroundings turned off. The system was then simulated for 5 ns, during which the PEs had their electrostatic and Lennard-Jones interactions linearly increased until they were fully interacting with the surrounding molecules at the end of the simulation. A position restraint along the *Z* dimension with a force constant of 1000 kJ mol^–1^ nm^–2^ was applied to the PE center of mass in order to prevent the PE from leaving its starting position during the early stages of the simulation. In the systems where multiple PEs are present, this procedure was performed sequentially for each type of PE. After all PEs had been inserted, the systems were equilibrated without restraints for 300 ns. This procedure for the insertion of PEs into the skin bilayer structure is summarized in Figure 1.

### Deuterium Order Parameters

The deuterium order parameters were calculated using the gmx order command from the GROMACS simulation package, with a selection of the ceramide sphingoid and fatty acid chain carbons, in separate command executions, that exist in all ceramides (fatty acids C2–C20 and sphingoid C4–C18). The first 50 ns of the simulation trajectories was discarded as equilibration.

## Results and Discussion

Modeling the permeability-enhancing effect of PEs is challenging, partly due to the complex nature of skin. Our approach is based on using MD simulations to calculate the free energy difference between a topical formulation and the skin’s barrier environment (Figure 1). This enables us to model the partitioning of formulation components into the barrier structure and then calculate their permeability-enhancing effect on various pharmaceuticals. Here, three major constraints need to be fulfilled: (1) the calculated permeability coefficients of a permeant in the absence of PEs should correlate with *in vitro*/*in vivo* measurements; (2) the molecular force field used in the MD simulations must be able to reproduce the partitioning behavior of all excipients, including the permeant and PEs, between the formulation and the skin’s barrier structure; (3) the calculated permeability enhancements should correlate with *in vitro*/*in vivo* measurements of a wide range of PEs and permeants.

We have previously shown that our model of the skin’s barrier structure, combined with the employed MD simulation techniques, is able to satisfy the first of these constraints, *i.e.*, that the calculated permeability coefficients of single permeants correlate with *in vitro*/*in vivo* measurements.^38^ To address the second constraint, *i.e.*, to verify that our chosen force field is able to adequately model the partitioning behavior of formulation excipients, we have now calculated the correlation between experimental and calculated excipient skin partition coefficients. Finally, as a first step toward addressing the third constraint, *i.e.*, the modeling of complex topical formulations including several PEs, we here chose to study saturated solutions of a single PE (using *in vitro* data from Pham et al.^23^) as well as ethanol/water formulations containing a single additional PE in low concentration (with data from Abd et al.^41^).

### Calculated Partition Coefficients

To assess the quality of the automated generation of small-molecule parameters with STaGE^61^ (and MATCH^63^), the octanol–water partition coefficients, log *K*_ow_, of 16 different molecules were calculated by combining the results from three different sets of solvation free energy calculations in water and octanol for each solute:
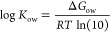
4where Δ*G*_ow_ is the difference in average solvation free energy in water compared to octanol, *R* is the gas constant, and *T* the temperature. The correlation with experimental values (Table S1 and Figure S1) gave an RMSE of 1.11 log units with *R*^2^ = 0.78, similar to previously reported log *K*_ow_ values for the CHARMM36 force field.^71^

**Figure 2:**
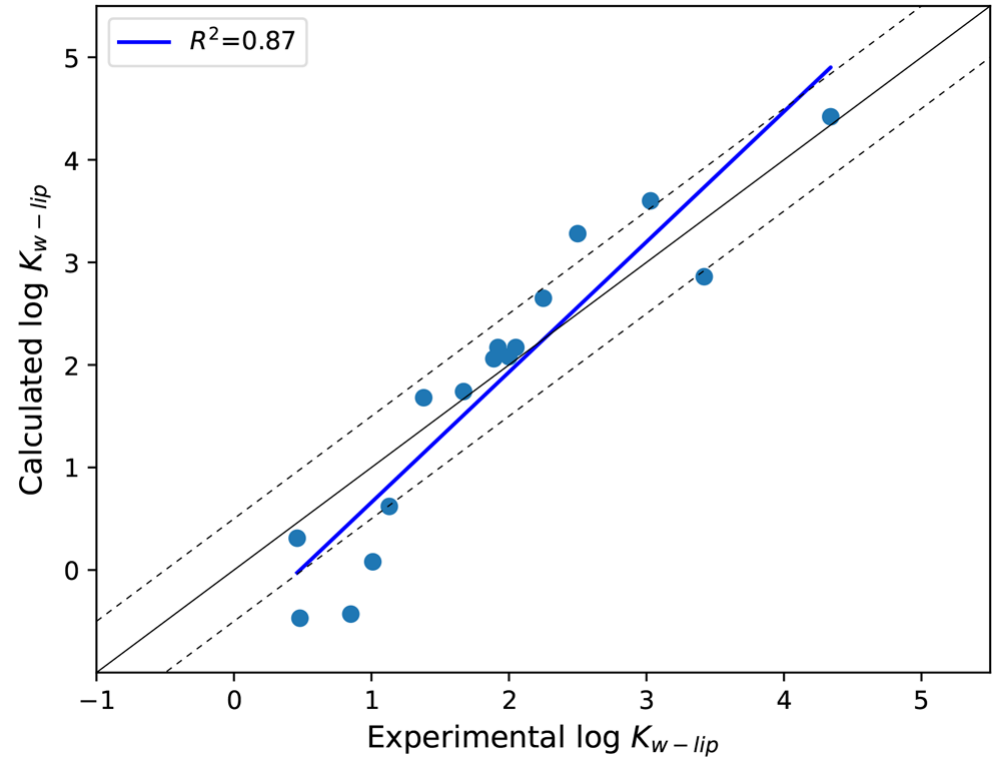
**Skin partition coefficients**. Correlation between calculated and experimentally measured partition coefficients from water into the barrier structure. Dashed lines above and below the identity line correspond to ±0.5 log unit. Data are also available in Table S1. Experimental data were taken from refs (18−20).

Following this, the partition coefficient from water into the skin’s barrier structure, log *K*_w–lip_ was calculated for all 16 molecules using eq 2, and the correlation with experimentally measured partition coefficients is displayed in Table S1 and Figure 2. The obtained RMSE was 0.58 log units with *R*^2^ = 0.87, indicating that the generated parameters seemed to adequately predict the partitioning behavior of small molecules into the skin’s barrier structure, with most of the outliers positioned around log *K*_w–lip_ < 1. The experimental relationship between log *K*_w–lip_ and log *K*_ow_ has previously been observed as^72^
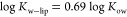
5which fits the *in vitro* data used here as well (Figure S2). For our calculated partition coefficients, the correlation is best fitted using a slope of 0.89.

### Construction of Skin Barrier Model Systems with Inserted Permeation Enhancers

**Figure 3:**
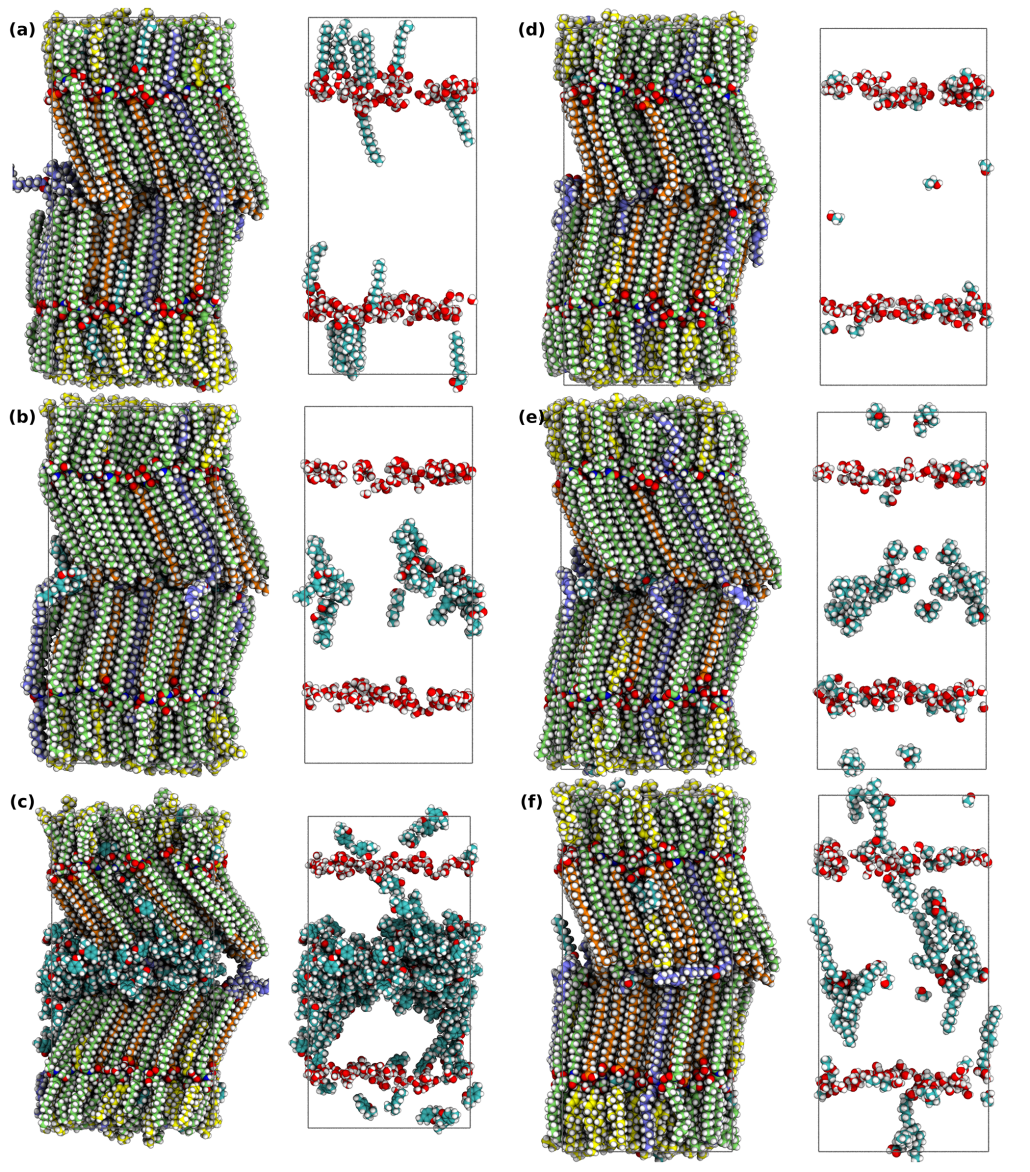
**Simulated skin barrier model systems with inserted permeation enhancers**. The skin’s barrier structure after insertion of permeability enhancers (PEs). The first column shows systems from ref (23) (Table 1): (a) water/lauric acid (16 inserted); (b) water/geraniol; (c) water/thymol. The second column shows systems from ref (41) (Table 2): (d) water/ethanol; (e) water/ethanol/eucalyptol; (f) water/ethanol/oleic acid (18 inserted). In each panel (a–f), the first figure shows the whole system, while the second figure shows the PE molecules and the water in the system. The carbon atoms are colored as follows: in nonacyl ceramides, green; in acyl ceramide EOS, light blue; in free fatty acids, orange; in cholesterol, yellow; in permeability enhancers, teal. The rest of the atoms are colored according to atom type (oxygen, red; nitrogen, blue; hydrogen, white).

The skin barrier model systems with inserted PEs are shown in Figure 3, and the calculated deuterium order parameters for the ceramides in the skin’s barrier structure are shown in Figure S3. We also calculated the number of hydrogen bonds between all the barrier lipids in each system; these are shown in Table S7. All excipients lower the number of hydrogen bonds compared to the pure skin system by approximately 15–30%. We have not observed any clear correlation between the amount of broken hydrogen bonds and permeability enhancement ratios for the permeants studied in this work.

The potential of mean force for each PE across the skin’s barrier structure is shown in Figure S4. The position along the lipid bilayer normal at which each PE is inserted is primarily decided by the lowest points along the PMF. Water and ethanol prefer to insert in the ceramide headgroup regions of the bilayer structure (Figure S4, *z* ∼ 3.2 nm). Eucalyptol, geraniol, and thymol prefer insertion close to the interface between either the sphingoid or fatty acid chains of the ceramides (Figure S4, *z* < 1 nm or *z* > 4.5 nm). Lauric acid, oleic acid, and stearic acid prefer to be inserted in the sphingoid or fatty acid chain regions of the ceramides (Figure S4, 1.5 < *z* < 2.5 nm or 4 < *z* < 4.5 nm).

Since the number of molecules to insert scales exponentially with the free energy difference between the formulation and the most favorable position in the lipid bilayer structure, we have obtained very high insertion numbers for some systems. In order to limit this effect, we currently use the calculated number of waters inserted from a pure water solution (see Table S2) as a guideline for the upper bound of the total mass of PEs to insert in the headgroup region. The calculated amount of water to insert (100 molecules) results in a total water content (190 molecules) that is roughly 2.1 times the water mass already present in the skin model (90 molecules), and we currently limit the insertion such that the total mass of PEs (including inserted additional water) in the headgroup region does not go above 2.5 times the mass of water already present in the system. The effect of this limit on the total number of molecules inserted in each simulated system is shown in Table S2 (note that only water and ethanol partition into the headgroup region—the other PEs are not affected). For the sphingoid and fatty acid tail regions, we have not found a suitable reference for an upper limit of insertion, and therefore, we do not limit the insertion in these regions.

### Metronidazole Skin Permeation Calculations

The calculated permeabilities for metronidazole, as well as comparisons with the experimental values obtained by Pham et al.,^23^ are displayed in Table 1. The skin barrier systems with inserted PEs are shown in the left column of Figure 3 (panels a–c), and the calculated permeation resistance profiles across the bilayer structure are shown in Figures S5–S7.

**Table 1:** *In Vitro* and Calculated Metronidazole Permeability Coefficients and the Corresponding Enhancement Ratios (ERs) for Different Saturated Water Formulations

	*in vitro*^a^		calculated^b^	
permeation enhancer	log *K*_P_ (cm h^–1^)	ER	log *K*_P_ (cm h^–1^)	ER
water	–2.89 ± 0.03	1	–3.79 ± 0.24	1
lauric acid (16 inserted)	–1.78 ± 0.07	13	–3.73 ± 0.23	1
lauric acid (24 inserted)			–2.97 ± 0.30	7
geraniol	–2.17 ± 0.04	5	–2.74 ± 0.21	11
stearic acid	–2.80 ± 0.03	1	–3.79 ± 0.24	1
thymol	> −1	>100	–1.39 ± 0.26	256

a Experimental data from Table 3 in ref (23).

b Calculated permeability coefficients are presented with one standard error of the mean.

Although calculated values for individual permeation enhancers in some cases deviate significantly from the corresponding measurements, the relative enhancement ratio (ER) compared reasonably well, with a slight underestimation of the absolute permeation enhancement. For stearic acid, the concentration in the formulation was so low (Table S2) that a negligible amount was predicted to partition into the skin’s barrier system during the permeation measurement of metronidazole. The calculated crossing time, from a pure water solution, for stearic acid was also 4 orders of magnitude longer than that of metronidazole. Thus, the system was assumed to be identical to that of the pure water phase since the stearic acid should have almost no influence on the solubility in the formulation or the permeation across the stratum corneum.

Lauric acid displayed a relatively large uncertainty in its PMF at the lowest points (see Figure S4 around 2.2–2.4 nm) which is the dominant region when calculating the partitioning. Based on the PMF, 16 molecules were to be inserted into the bilayer structure, but this resulted in almost no difference in observed permeability, in disagreement with the experimental data. Therefore, we decided to increase the amount of inserted lauric acid molecules in a stepwise manner, increasing the amount by 50% at each step. Snapshots of the equilibrated systems are shown in Figure S8. From the series of simulations with increasing amounts of lauric acid, we observe a fast increase of the permeability enhancement already when inserting 50% additional lauric acid (24 inserted), as shown in Table 1. When the amount is increased further, no statistically significant extra enhancement is observed (see Table S3).

From the reference permeation resistance profile for metronidazole (*e.g.*, black curve in Figure S5), we can see the that the main permeability barrier is situated in the ceramide sphingoid chain region (4 nm < *z* < 5 nm), with the headgroup region (*z* ∼ 3.2 nm) posing the least resistance followed by a second barrier in the ceramide fatty acid chain region (*z* ∼ 2.1 nm). When inserting lauric acid into the skin bilayer (Figure S5), we see a reduction of the barrier in the ceramide sphingoid chain region of the bilayer stucture with increasing lauric acid content. The barrier in the ceramide fatty acid chain region increases with 16 inserted lauric acids but is otherwise relatively unchanged compared to the reference.

For geraniol we found multiple values reported in the literature regarding its solubility in pure water: 100 mg L^–1^ (at 298 K),^73,74^ 229 mg L^–1^ (no temperature reported),^75^ and 686 mg L^–1^ (at 293 K).^76^ For completeness, we decided to calculate the effect of three different formulations of geraniol: 100, 200, and 686 mg L^–1^ (all permeability values are reported in Table S3). The solubility series reported recently by Martins et al.^74^ indicates that the most likely correct water solubility of geraniol at a temperature of 305 K would be roughly 200 mg L^–1^, which is the formulation used to calculate the data we report in Table 1. From Table S3 we see that the permeability enhancement mimics the solubilities, with the highest solubility having the highest permeation enhancement. The geraniol molecules prefer to aggregate in the fatty acid chain region of the skin’s barrier structure (Figure S9) but there is no apparent effect on the permeation resistance in this region for the two lower-solubility calculations (Figure S6). Instead, there is a clear reduction of the barrier capacity in the ceramide sphingoid chain region, most likely related to the decreased lipid ordering in this part of the system (Figure S3a).

For thymol, when using the saturated concentration of 900 mg L^–1^, the partitioning calculations resulted in a local concentration (in the most populated insertion slice in the lipid bilayer structure) of thymol that would be higher than that of a pure thymol solution, and the resulting amount of inserted thymol was roughly equal to the mass of the whole bilayer (see Table S2 and Figure S10). Since this concentration would be unrealistic, we also performed calculations with lower concentrations of thymol in the skin’s barrier structure. The concentration of pure liquid thymol is 3.9 molecules nm^–3^ (density 0.97 g cm^–3^) and we constructed two systems in which the highest local concentration was either 2.3 molecules nm^–3^ or 4.7 molecules nm^–3^. These local concentrations corresponded to a calculated thymol concentration in the formulation of either 100 or 200 mg L^–1^, which is how they are denoted in Table S2, S3, S5 and Figures S3, S7, S10. The data presented in Table 1 are based on the system corresponding to a thymol concentration of 200 mg L^–1^ in the formulation. In the calculated permeation resistance profiles (Figure S7) we see a large reduction of the permeability barrier for all the simulated thymol systems, with an almost complete removal of the barrier for the system with a thymol concentration of 900 mg L^–1^ in the formulation (as expected given the unrealistic thymol concentration in the system). The *in vitro* measurement never reached steady state, indicating a very high permeability enhancement for thymol, which is also reflected in the calculated values.

### Caffeine and Naproxen Skin Permeation Calculations

The calculated permeabilities for caffeine and naproxen, as well as comparisons with the experimental values obtained by Abd et al.,^41^ are displayed in Table 2. The skin barrier model systems with inserted PEs are shown in the right column of Figure 3 (panels d–f), and the calculated permeation resistance profiles across the lipid bilayer structure are shown in Figure S11. When calculating the partitioning from the formulations into the skin’s barrier structure, there is a need to account for ethanol evaporation in the formulations. A correct estimate of ethanol evaporation during formulation preparation, subsequent formulation skin application, and final permeability measurement (even within covered donor chambers some evaporation occurs) is difficult to assess.^77,78^ For these calculations, we have assumed an ethanol evaporation of 50% from the formulations, but a more in-depth evaluation is envisaged for future studies. Furthermore, since ionization of naproxen (p*K*_a_ = 4.19) varies between simulated formulations (pH between 2.9 and 4.8^41^), the naproxen permeability coefficients were shifted based on the formulation pH according to
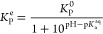
6where *K*_P_^e^ is the effective permeability coefficient, *K*_P_^0^ is the calculated permeability coefficient, and p*K*_a_^aq^ is the aqueous p*K*_a_ of naproxen. This shift results in a better agreement in absolute permeation coefficients as well as in *in vitro* enhancement ratios for naproxen (the unmodified and pH-corrected permeability coefficients can be seen in Table S4). Similarly to metronidazole, the reference resistance profile for caffeine (black curve in Figure S11) has its main permeability barrier in the ceramide sphingoid chain region (*z* ∼ 4.8 nm), with a minimum in the headgroup region followed by a second barrier in the ceramide fatty acid chain region (1 nm < *z* < 2 nm). For naproxen (note that the reference simulation is from an ethanol/water formulation), a major permeability barrier can be seen in the ceramide sphingoid chain region around *z* ∼ 4.8 nm, similar to caffeine. Contrary to caffeine, naproxen then experiences a second barrier close to the headgroup region (*z* ∼ 3.5 nm) and possibly a minor third barrier around *z* ∼ 1.3 nm in the ceramide fatty acid region.

**Table 2:** *In Vitro* and Calculated Permeability Coefficients for Caffeine and Naproxen

	*in vitro*^a^				calculated^b^	
PE	*J*_max_ (μg cm^–2^ h^–1^)	ER^c^	log *K*_P_ (cm h^–1^)	ER	log *K*_P_ (cm h^–1^)	ER
Caffeine Permeability						
water	2.2 ± 0.6		–4.15 ± 0.04	1.0	–3.00 ± 0.21	1
water/ethanol^d^	25.6 ± 2.5	4.9	–3.50 ± 0.02	4.5	–2.28 ± 0.24	5
oleic acid (18 inserted)^e^	692.6 ± 24.0	132.5	–1.91 ± 0.02	175	–1.92 ± 0.18	12
oleic acid (36 inserted)^e^					–2.21 ± 0.20	6
eucalyptol (26 inserted)^f^	1015.8 ± 28.9	194.3	–1.99 ± 0.01	148	–2.64 ± 0.23	2
eucalyptol (52 inserted)^f^					–2.00 ± 0.25	10
Naproxen Permeability						
water/ethanol^d^	23.4 ± 3.9	1.0	–3.34 ± 0.07	1.0	–2.85 ± 0.10	1
oleic acid (18 inserted)^e^	446.3 ± 37.9	18.9	–1.73 ± 0.02	41	–2.39 ± 0.13	3
oleic acid (36 inserted)^e^					–1.68 ± 0.11	15
eucalyptol (26 inserted)^f^	74.9 ± 12.3	3.2	– 2.44 ± 0.04	7.9	–2.46 ± 0.11	3
eucalyptol (52 inserted)^f^					–2.02 ± 0.17	7

a Experimental data were taken from Tables 1 and 2 in ref (41).

b Calculated permeability coefficients are presented with one standard error of the mean.

c Caffeine enhancement ratios were taken from Table 2 in ref (41).

d 40% water, 60% ethanol.

e 40% water, 60% ethanol, 3% w/v oleic acid.

f 40% water, 60% ethanol, 5% w/v eucalyptol.

The addition of 60% ethanol to a water solution lowers the main permeability barrier of caffeine in the ceramide sphingoid chain region (Figure S11) but has almost no effect in other parts of the system. For both caffeine and naproxen we see an increased permeation resistance in the fatty acid chain region (*z* ∼ 1.8 for caffeine and 1.5 < *z* < 3 nm for naproxen) in the systems with inserted eucalyptol, while only a small or no reduction of the barrier capacity is seen in the ceramide sphingoid chain region. Since the permeability enhancement of eucalyptol was much lower than expected, we also performed calculations with twice the amount of eucalyptol inserted (Figure S12), which in both cases resulted in a higher permeability enhancement and thus better agreement with experimental data.

The calculated permeability of naproxen through the systems with inserted oleic acid displayed a very low increase in permeability (similar to metronidazole with lauric acid as a PE) compared to the *in vitro* data. Therefore, we performed a second iteration of the skin partitioning calculations, using the first constructed version of the system (Figure S13a) to recalculate the PMF of oleic acid. The PMFs from both iterations are shown in Figure S14, and the final inserted amount of oleic acid was 54 molecules (Figure S13c). In a similar fashion to the simulations with lauric acid, we decided to increase the amount of inserted oleic acid in steps, going from 18 to 36 and then finally to 54 inserted molecules. The permeation resistance profiles (Figure S11) of caffeine and naproxen calculated across the different systems with oleic acid show a decrease of the permeation resistance in the ceramide sphingoid chain region (4.7 nm < *z* < 5.2 nm) for all systems. The permeation resistance in the ceramide fatty acid chain region for caffeine appears relatively unchanged, except for the system with 18 inserted oleic acid molecules, in which the barrier capacity decreases slightly. For naproxen the barriers close to the ceramide headgroup region and in the ceramide fatty acid chain region both increase with the insertion of 18 oleic acid molecules, but for 36 and 54 inserted molecules the barrier in the ceramide headgroup region is reduced and the barrier in the ceramide fatty acid chain region is relatively unchanged.

For the calculated permeability values, starting with caffeine, we observe a relatively good qualitative agreement between the *in vitro* measurements and the calculated ERs. However, the large increase in the *in vitro* permeability enhancement from both oleic acid and eucalyptol is greatly reduced in the calculated data. It should be noted that the caffeine permeability coefficient reported by Abd et al. (−4.15) is lower than those reported elsewhere (*e.g.*, −3.6^79^ and −3.2^80^). Unfortunately, caffeine has been reported to be one of the more problematic small molecules to parametrize correctly in existing force fields,^81,82^ and the CHARMM36 force field parameters used in these simulations overestimate the log *K*_ow_ of caffeine by 2 log units (see Table S1). This might be an explanation for the poor permeation enhancement obtained in the simulations since a lipophilic molecule experiences lower permeation barriers across the skin’s lipid bilayer structure. Furthermore, our obtained standard errors in the calculated permeability coefficients for caffeine are so large that they cannot be deemed statistically different, just higher than that of the reference.

For naproxen, we see relatively good agreement between the calculated permeability coefficients and ERs compared to the *in vitro* data. When compared to the ERs based on *in vitro* permeability coefficients, the calculated ERs for oleic acid and eucalyptol are on the lower side although still within the same order of magnitude (and relative difference) as the *in vitro* data. The ERs based on *in vitro* maximum flux compare very well with the calculated data with an additional amount of inserted permeability enhancers. This trend is consistent for both PEs, *i.e.*, we see a low permeability enhancement using the amount of inserted molecules suggested by eq 3, but the simulations with an increased amount have a better correlation with *in vitro* data. This indicates that the current methodology might underestimate the amount of PEs that should be inserted into the system.

### Flux Optimization Using the Potential of Mean Force

The flux of a permeant across the skin’s barrier, *J*, is calculated as
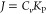
7where *C*_*v*_ is the permeant concentration in the formulation and *K*_P_ is the permeability coefficient. Since the PMF across the skin’s barrier structure is calibrated against the solvation free energy of the permeant in the formulation (Δ*G*_transfer_(*z*) in eq 1), it should be possible to use the minimum in the calculated PMFs to optimize the permeant flux from a specific formulation. For optimal flux, the permeant free energy difference between a formulation and the skin’s barrier structure (*i.e.*, the difference between the solvation free energy in the formulation and the minimum of the PMF) should be close to zero, as this enables a high solubility of the permeant in the formulation (thus maximizing its concentration) while maintaining a good partitioning of the permeant into the skin’s barrier structure. Thus, depending on where along the *y* axis (see Figure S15) the PMF minimum of a permeant is located, we would argue that two different methods to modify the permeant’s flux across the barrier exist. In the first case, when the PMF minimum is below zero, the solubility of the permeant in the formulation can be increased without modifying the *K*_P_ (see eq 1 and the paragraph just after the equation). In such cases, one could modify the formulation using inert excipients that have no impact on the skin’s barrier structure (*e.g.*, long-chain PEG or Brij 98) in order to increase the concentration of the permeant in the formulation and thus increase its flux across the barrier. In the second case, when the PMF minimum is above zero, an increased permeant solubility in the formulation would increase the PMF and thus result in a reduced *K*_P_. In this situation, there would be no positive effect on the permeant flux by increasing the permeant’s solubility in the formulation, since an increased formulation solubility would result in a similar increase in the relative permeation barriers across the skin’s barrier structure. Instead, focus should be on modifying the formulation using PEs that reduce the permeant’s permeation barriers across the barrier structure.

As an example, naproxen has a reported maximal flux of 0.6 μg cm^–2^ h^–1^ from water,^83^ and its PMF minimum from a water solution in Figure S15 is −5.4 ± 1.0 kJ mol^–1^. If one were to add, *e.g.*, Brij 98 to the water solution (increasing the naproxen solubility and thus maximizing its concentration), raising the PMF minimum to zero, one would expect the maximum flux to be ∼10 times higher, which is similar to what Abd et al. reported for their measurements of naproxen permeability when varying the solubility using different inert control vehicles.^41^ For their PEG6000/water formulations, they saw a plateau of the permeability enhancement at a solubility of 45 mg cm^–3^, at which they reached a maximal flux of roughly 7 μg cm^–2^ h^–1^, thus obtaining a maximal enhancement ratio (ER) of approximately 10 compared to naproxen from pure water. For caffeine their data show a similar concentration-dependent behavior of the flux (an initial increase from 2.2 μg cm^–2^ h^–1^ until a plateau in flux values is reached around 6.6 μg cm^–2^ h^–1^),^41^ but our calculated value for the PMF minimum is roughly −13 ± 1.0 kJ mol^–1^, which is a clear overestimation (corresponding to a possibility to increase the flux roughly 180 times) of the partitioning compared to the *in vitro* data. Given that the force field parameters for caffeine appear to be slightly too lipophilic (log *K*_ow_ in Table S1), we would expect the real partitioning free energy to be much higher.

The authors are aware of the fact that this idea goes against conventional theory regarding permeability from saturated solutions (that maximum flux is independent of the solubility in saturated inert solutions that do not affect the skin barrier) but still believe that it would be of interest to test this in controlled steady-state skin permeability experiments in the future.

## 4 Conclusions

Understanding how chemical permeation enhancers (PEs) impact skin to improve transdermal delivery of drugs has great implications for clinical medicine, as replacing oral or intravenous drug administration with topical drug administration would give physicians better control over medication and dosage levels. Due to the inability of current *in vitro* methods to predict the effects of PEs on transdermal drug transport, complementary *in silico* methods are requested. We have presented our current approach using molecular dynamics (MD) simulations to predict the effect of PEs on drug permeation through skin. The atomistic resolution of MD simulations allows a detailed description of the permeability barriers in skin and enables investigation of how PEs modify these barriers at different locations across the skin’s barrier structure. Qualitative agreement between calculated and *in vitro*-determined permeability coefficients is good, and MD simulations are able to reproduce permeability enhancement ratio (ER) rankings. This indicates that the fundamental models, simulations, and sampling algorithms used are sound, and with smaller statistical errors we are able to identify both outliers and systematic limitations of the models.

Still, the quantiative correlation of the calculated enhancement ratios with *in vitro* data needs further refinement, with future work focusing on modeling formulation excipient evaporation, permeant ionization in relation to pH, permeation speed of PEs in relation to permeant permeation speed, and skin partitioning of formulation excipients. Some excipients or permeants, such as caffeine, may be difficult to model using the CHARMM36 molecular force field. However, a preliminary screen of molecular properties (*e.g.*, octanol–water partition coefficients) might give an indication of such cases.

Calculated permeant potentials of mean force across the skin’s barrier structure can be used to determine whether modification of a permeant’s permeation barriers in skin with PEs or modification of its solubility in the formulation will be most efficient to increase the flux of a permeant across skin.

Future refinement of the calculation of formulation excipient partitioning into skin might be called for, as our current setup appears to underestimate the excipient amounts inserted. Improvements regarding the incorporation of long-chain fatty acids, or other lipids, into the skin’s barrier structure may also be requested. Although the use of the pH – p*K*_a_ difference to shift permeability coefficients appears to work well for charged molecules, a correct treatment of ionization states and salts might require a more advanced simulation protocol such as constant-pH simulations.^84^

## Data and Software Availability

The input data and parameters are available for download at 10.5281/zenodo.7620240.

## Supporting Information Available

Calculated partition coefficients between water and the skin’s barrier structure; number of inserted molecules from each formulation modeled; calculated permeability coefficients; molecular compositions of simulated formulations; calculated octanol–water partition coefficients; calculated deuterium order parameters; potentials of mean force for each permeability enhancer; potentials of mean force and permeation resistance profiles for naproxen, caffeine, and metronidazole; and skin barrier structures after insertion of permeation enhancers (PDF)

## Footnotes

a Relative composition in mol % ceramides/mol % cholesterol/mol % free fatty acids/relative amount of cholesterol on ceramide sphingoid side/mol % acyl ceramide EOS (included in the relative ceramide concentration)/water molecules per lipid (not included in the mol % concentrations of the lipids).

## References

[ref1] Breathnach A. S.; Goodman T.; Stolinski C.; Gross M. Freeze-fracture replication of cells of stratum corneum of human epidermis. J. Anat. 1973, 114, 65–81. 4736654 PMC1271426

[ref2] Elias P. M.; Friend D. S. The permeability barrier in mammalian epidermis. J. Cell Biol. 1975, 65, 180–191. 10.1083/jcb.65.1.180. 1127009 PMC2111161

[ref3] Elias P.; Feingold K.; Fluhr J. In Fitzpatrick’s Dermatology in General Medicine, 6th ed.; Freedberg I. M., Eisen A. Z., Wolff K., Austen K. F., Goldsmith L. A., Katz S. I., Eds.; McGraw Hill: New York, 2003; Vol. 1, Chap. 9; 107 118.

[ref4] Wertz P. W.; Kremer M.; Squier C. A. Comparison of lipids from epidermal and palatal stratum corneum. J. Invest. Dermatol. 1992, 98, 375–8. 10.1111/1523-1747.ep12499809. 1545146

[ref5] Law S.; Wertz P. W.; Swartzendruber D. C.; Squier C. A. Regional variation in content, composition and organization of porcine epithelial barrier lipids revealed by thin-layer chromatography and transmission electron microscopy. Arch. Oral Biol. 1995, 40, 1085–91. 10.1016/0003-9969(95)00091-7. 8850646

[ref6] Madison K. C. Barrier function of the skin: ”la raison d’être” of the epidermis. J. Invest Dermatol. 2003, 121, 231–241. 10.1046/j.1523-1747.2003.12359.x. 12880413

[ref7] Bouwstra J. A.; de Graaff A.; Gooris G. S.; Nijsse J.; Wiechers J. W.; van Aelst A. C. Water Distribution and Related Morphology in Human Stratum Corneum at Different Hydration Levels. J. Invest. Dermatol. 2003, 120, 750–758. 10.1046/j.1523-1747.2003.12128.x. 12713576

[ref8] Lundborg M.; Narangifard A.; Wennberg C. L.; Lindahl E.; Daneholt B.; Norlén L. Human skin barrier structure and function analyzed by cryo-EM and molecular dynamics simulation. J. Struct. Biol. 2018, 203, 149–161. 10.1016/j.jsb.2018.04.005. 29702212

[ref9] Swartzendruber D. C.; Wertz P. W.; Kitko D. J.; Madison K. C.; Downing D. T. Molecular models of the Intercellular Lipid Lamellae in Mammalian Stratum Corneum. J. Invest. Dermatol. 1989, 92, 251–257. 10.1111/1523-1747.ep12276794. 2918233

[ref10] Bouwstra J.; Pilgram G.; Gooris G.; Koerten H.; Ponec M. New Aspects of the Skin Barrier Organization. Skin Pharmacol. Appl. Skin Physiol. 2001, 14, 52–62. 10.1159/000056391. 11509908

[ref11] Hill J. R.; Wertz P. W. Molecular models of the intercellular lipid lamellae from epidermal stratum corneum. Biochim. Biophys. Acta, Biomembr. 2003, 1616, 121–126. 10.1016/S0005-2736(03)00238-4. 14561469

[ref12] McIntosh T. J. Organization of Skin Stratum Corneum Extracellular Lamellae: Diffraction Evidence for Asymmetric Distribution of Cholesterol. Biophys. J. 2003, 85, 1675–1681. 10.1016/S0006-3495(03)74597-4. 12944282 PMC1303341

[ref13] Schröter A.; Kessner D.; Kiselev M. A.; Hauß T.; Dante S.; Neubert R. H. H. Basic Nanostructure of Stratum Corneum Lipid Matrices Based on Ceramides [EOS] and [AP]: A Neutron Diffraction Study. Biophys. J. 2009, 97, 1104–1114. 10.1016/j.bpj.2009.05.041. 19686658 PMC2726320

[ref14] Iwai I.; Han H.; Hollander L. d.; Svensson S.; Öfverstedt L.-G.; Anwar J.; Brewer J.; Bloksgaard M.; Laloeuf A.; Nosek D.; Masich S.; Bagatolli L. A.; Skoglund U.; Norlén L. The Human Skin Barrier Is Organized as Stacked Bilayers of Fully Extended Ceramides with Cholesterol Molecules Associated with the Ceramide Sphingoid Moiety. J. Invest. Dermatol. 2012, 132, 2215–2225. 10.1038/jid.2012.43. 22534876

[ref15] Mojumdar E. H.; Gooris G. S.; Groen D.; Barlow D. J.; Lawrence M. J.; Demé B.; Bouwstra J. A. Stratum corneum lipid matrix: location of acyl ceramide and cholesterol in the unit cell of the long periodicity phase. Biochim. Biophys. Acta, Biomembr. 2016, 1858, 1926–1934. 10.1016/j.bbamem.2016.05.006. 27169629

[ref16] Bartosova L.; Bajgar J. Transdermal Drug Delivery In Vitro Using Diffusion Cells. Curr. Med. Chem. 2012, 19, 4671–4677. 10.2174/092986712803306358. 22934776

[ref17] Williams A. C.; Barry B. W. Penetration enhancers. Adv. Drug Delivery Rev. 2012, 64, 128–137. 10.1016/j.addr.2012.09.032. 15019749

[ref18] Johnson M. E.; Blankschtein D.; Langer R. Evaluation of solute permeation through the stratum corneum: lateral bilayer diffusion as the primary transport mechanism. J. Pharm. Sci. 1997, 86, 1162–1172. 10.1021/js960198e. 9344175

[ref19] Hansen S.; Henning A.; Naegel A.; Heisig M.; Wittum G.; Neumann D.; Kostka K.-H.; Zbytovska J.; Lehr C.-M.; Schaefer U. F. In-silico model of skin penetration based on experimentally determined input parameters. Part I: experimental determination of partition and diffusion coefficients. Eur. J. Pharm. Biopharm. 2008, 68, 352–367. 10.1016/j.ejpb.2007.05.012. 17587558

[ref20] Ellison C. A.; Tankersley K. O.; Obringer C. M.; Carr G. J.; Manwaring J.; Rothe H.; Duplan H.; Géniès C.; Grégoire S.; Hewitt N. J.; Jamin C. J.; Klaric M.; Lange D.; Rolaki A.; Schepky A. Partition coefficient and diffusion coefficient determinations of 50 compounds in human intact skin, isolated skin layers and isolated stratum corneum lipids. Toxicol. In Vitro 2020, 69, 104990 10.1016/j.tiv.2020.104990. 32882340

[ref21] Potts R. O.; Golden G. M.; Francoeur M. L.; Mak V. H. W.; Guy R. H. Mechanism and enhancement of solute transport across the stratum corneum. J. Controlled Release 1991, 15, 249–260. 10.1016/0168-3659(91)90116-U.

[ref22] Cornwell P. A.; Barry B. W.; Bouwstra J. A.; Gooris G. S. Modes of action of terpene penetration enhancers in human skin; Differential scanning calorimetry, small-angle X-ray diffraction and enhancer uptake studies. Int. J. Pharm. 1996, 127, 9–26. 10.1016/0378-5173(95)04108-7.

[ref23] Pham Q. D.; Björklund S.; Engblom J.; Topgaard D.; Sparr E. Chemical penetration enhancers in stratum corneum—Relation between molecular effects and barrier function. J. Controlled Release 2016, 232, 175–187. 10.1016/j.jconrel.2016.04.030. 27108613

[ref24] Potts R. O.; Guy R. H. Predicting Skin Permeability. Pharm. Res. 1992, 9, 663–669. 10.1023/A:1015810312465. 1608900

[ref25] Mitragotri S. A theoretical analysis of permeation of small hydrophobic solutes across the stratum corneum based on Scaled Particle Theory. J. Pharm. Sci. 2002, 91, 744–752. 10.1002/jps.10048. 11920759

[ref26] Abraham M. H.; Martins F. Human skin permeation and partition: General linear free-energy relationship analyses. J. Pharm. Sci. 2004, 93, 1508–1523. 10.1002/jps.20070. 15124209

[ref27] Tsakovska I.; Pajeva I.; Al Sharif M.; Alov P.; Fioravanzo E.; Kovarich S.; Worth A. P.; Richarz A.-N.; Yang C.; Mostrag-Szlichtyng A.; Cronin M. T. Quantitative structure-skin permeability relationships. Toxicology 2017, 387, 27–42. 10.1016/j.tox.2017.06.008. 28645577

[ref28] Lian G.; Chen L.; Han L. An evaluation of mathematical models for predicting skin permeability. J. Pharm. Sci. 2008, 97, 584–598. 10.1002/jps.21074. 17722002

[ref29] Höltje M.; Förster T.; Brandt B.; Engels T.; von Rybinski W.; Höltje H.-D. Molecular dynamics simulations of stratum corneum lipid models: fatty acids and cholesterol. Biochim. Biophys. Acta, Biomembr. 2001, 1511, 156–167. 10.1016/S0005-2736(01)00270-X. 11248214

[ref30] Notman R.; den Otter W. K.; Noro M. G.; Briels W. J.; Anwar J. The Permeability Enhancing Mechanism of DMSO in Ceramide Bilayers Simulated by Molecular Dynamics. Biophys. J. 2007, 93, 2056–2068. 10.1529/biophysj.107.104703. 17513383 PMC1959535

[ref31] Das C.; Olmsted P. D.; Noro M. G. Water permeation through stratum corneum lipid bilayers from atomistic simulations. Soft Matter 2009, 5, 4549–4555. 10.1039/b911257j.

[ref32] Gupta R.; Sridhar D. B.; Rai B. Molecular Dynamics Simulation Study of Permeation of Molecules through Skin Lipid Bilayer. J. Phys. Chem. B 2016, 120, 8987–8996. 10.1021/acs.jpcb.6b05451. 27518707

[ref33] Rocco P.; Cilurzo F.; Minghetti P.; Vistoli G.; Pedretti A. Molecular Dynamics as a tool for in silico screening of skin permeability. Eur. J. Pharm. Sci. 2017, 106, 328–335. 10.1016/j.ejps.2017.06.020. 28627472

[ref34] Del Regno A.; Notman R. Permeation pathways through lateral domains in model membranes of skin lipids. Phys. Chem. Chem. Phys. 2018, 20, 2162–2174. 10.1039/C7CP03258G. 29116267

[ref35] Gupta R.; Dwadasi B. S.; Rai B.; Mitragotri S. Effect of Chemical Permeation Enhancers of Skin Permeability: *In silico* screening using Molecular Dynamics simulations. Sci. Rep. 2019, 9, 1456 10.1038/s41598-018-37900-0. 30728438 PMC6365548

[ref36] Wang E.; Klauda J. B. Molecular Structure of the Long Periodicity Phase in the Stratum Corneum. J. Am. Chem. Soc. 2019, 141, 16930–16943. 10.1021/jacs.9b08995. 31547662

[ref37] MacDermaid C. M.; Hall K. W.; DeVane R. H.; Klein M. L.; Fiorin G. Coexistence of Lipid Phases Stabilizes Interstitial Water in the Outer Layer of Mammalian Skin. Biophys. J. 2020, 118, 1588–1601. 10.1016/j.bpj.2020.01.044. 32101711 PMC7136285

[ref38] Lundborg M.; Wennberg C.; Lidmar J.; Hess B.; Lindahl E.; Norlén L. Skin permeability prediction with MD simulation sampling spatial and alchemical reaction coordinates. Biophys. J. 2022, 121, 3837–3849. 10.1016/j.bpj.2022.09.009. 36104960 PMC9674988

[ref39] Scheuplein R. J.; Blank I. H.; Brauner G. J.; Macfarlane D. J. Percutaneous Absorption of Steroids. J. Invest. Dermatol. 1969, 52, 63–70. 10.1038/jid.1969.9. 5761930

[ref40] Sznitowska M.; Janicki S.; Baczek A. Studies on the effect of pH on the lipoidal route of penetration across stratum corneum. J. Controlled Release 2001, 76, 327–335. 10.1016/S0168-3659(01)00443-6. 11578746

[ref41] Abd E.; Benson H. A.; Mohammed Y. H.; Roberts M. S.; Grice J. E. Permeation Mechanism of Caffeine and Naproxen htrough in vitro Human Epidermis: Effect of Vehicles and Penetration Enhancers. Skin Pharmacol. Physiol. 2019, 32, 132–141. 10.1159/000497225. 30909278

[ref42] Lundborg M.; Lidmar J.; Hess B. The accelerated weight histogram method for alchemical free energy calculations. J. Chem. Phys. 2021, 154, 204103 10.1063/5.0044352. 34241154

[ref43] Abraham M. J.; Murtola T.; Schulz R.; Páll S.; Smith J. C.; Hess B.; Lindahl E. GROMACS: High performance molecular simulations through multi-level parallelism from laptops to supercomputers. SoftwareX 2015, 1–2, 19–25. 10.1016/j.softx.2015.06.001.

[ref44] Lindahl E.; Abraham M. J.; Hess B.; van der Spoel D. GROMACS 2021 Manual., 2021. 10.5281/zenodo.4457591 (accessed 2021-03-05).

[ref45] Páll S.; Zhmurov A.; Bauer P.; Abraham M.; Lundborg M.; Gray A.; Hess B.; Lindahl E. Heterogeneous parallelization and acceleration of molecular dynamics simulations in GROMACS. J. Chem. Phys. 2020, 153, 134110 10.1063/5.0018516. 33032406

[ref46] Lidmar J. Improving the efficiency of extended ensemble simulations: The accelerated weight histogram method. Phys. Rev. E 2012, 85, 056708 10.1103/PhysRevE.85.056708. 23004904

[ref47] Lindahl V.; Lidmar J.; Hess B. Accelerated weight histogram method for exploring free energy landscapes. J. Chem. Phys. 2014, 141, 044110 10.1063/1.4890371. 25084884

[ref48] GROMACS gitlab: 2022-awhsymm-awhcorrblocks, 2022. https://gitlab.com/gromacs/gromacs/-/tree/2022-awhsymm-awhcorrblocks (accessed 2021-11-19).

[ref49] Lundborg M.; Wennberg C. L.; Narangifard A.; Lindahl E.; Norlén L. Predicting drug permeability through skin using molecular dynamics simulation. J. Controlled Release 2018, 283, 269–279. 10.1016/j.jconrel.2018.05.026. 29864475

[ref50] Essmann U.; Perera L.; Berkowitz M. L.; Darden T.; Lee H.; Pedersen L. G. A smooth particle mesh Ewald method. J. Chem. Phys. 1995, 103, 8577–8593. 10.1063/1.470117.

[ref51] Hess B.; Bekker H.; Berendsen H. J. C.; Fraaije J. G. E. M. LINCS: A linear constraint solver for molecular simulations. J. Comput. Chem. 1997, 18, 1463–1472. 10.1002/(SICI)1096-987X(199709)18:12<1463::AID-JCC4>3.0.CO;2-H.

[ref52] Miyamoto S.; Kollman P. A. Settle: An analytical version of the SHAKE and RATTLE algorithm for rigid water models. J. Comput. Chem. 1992, 13, 952–962. 10.1002/jcc.540130805.

[ref53] Jorgensen W. L.; Chandrasekhar J.; Madura J. D.; Impey R. W.; Klein M. L. Comparison of simple potential functions for simulating liquid water. J. Chem. Phys. 1983, 79, 926–935. 10.1063/1.445869.

[ref54] Klauda J. B.; Venable R. M.; Freites J. A.; O’Connor J. W.; Tobias D. J.; Mondragon-Ramirez C.; Vorobyov I.; MacKerell A. D.; Pastor R. W. Update of the CHARMM All-Atom Additive Force Field for Lipids: Validation on Six Lipid Types. J. Phys. Chem. B 2010, 114, 7830–7843. 10.1021/jp101759q. 20496934 PMC2922408

[ref55] Venable R.; Sodt A.; Rogaski B.; Rui H.; Hatcher E.; MacKerell A. Jr.; Pastor R.; Klauda J. CHARMM All-Atom Additive Force Field for Sphingomyelin: Elucidation of Hydrogen Bonding and of Positive Curvature. Biophys. J. 2014, 107, 134–145. 10.1016/j.bpj.2014.05.034. 24988348 PMC4119286

[ref56] Dahlén B.; Pascher I. Molecular arrangements in sphingolipids. Thermotropic phase behaviour of tetracosanoylphytosphingosine. Chem. Phys. Lipids 1979, 24, 119–133. 10.1016/0009-3084(79)90082-3.

[ref57] Feenstra K. A.; Hess B.; Berendsen H. J. C. Improving efficiency of large time-scale molecular dynamics simulations of hydrogen-rich systems. J. Comput. Chem. 1999, 20, 786–798. 10.1002/(SICI)1096-987X(199906)20:8<786::AID-JCC5>3.0.CO;2-B. 35619462

[ref58] Hopkins C. W.; Le Grand S.; Walker R. C.; Roitberg A. E. Long-Time-Step Molecular Dynamics through Hydrogen Mass Repartitioning. J. Chem. Theory Comput. 2015, 11, 1864–1874. 10.1021/ct5010406. 26574392

[ref59] Bernetti M.; Bussi G. Pressure control using stochastic cell rescaling. J. Chem. Phys. 2020, 153, 114107 10.1063/5.0020514. 32962386

[ref60] Beutler T. C.; Mark A. E.; van Schaik R. C.; Gerber P. R.; van Gunsteren W. F. Avoiding singularities and numerical instabilities in free energy calculations based on molecular simulations. Chem. Phys. Lett. 1994, 222, 529–539. 10.1016/0009-2614(94)00397-1.

[ref61] Lundborg M.; Lindahl E. Automatic GROMACS Topology Generation and Comparisons of Force Fields for Solvation Free Energy Calculations. J. Phys. Chem. B 2015, 119, 810–823. 10.1021/jp505332p. 25343332

[ref62] O’Boyle N. M.; Banck M.; James C. A.; Morley C.; Vandermeersch T.; Hutchison G. R. Open Babel: An open chemical toolbox. J. Cheminf. 2011, 3, 33 10.1186/1758-2946-3-33. PMC3198950 21982300

[ref63] Yesselman J. D.; Price D. J.; Knight J. L.; Brooks C. L. III. MATCH: an atom-typing toolset for molecular mechanics force fields. J. Comput. Chem. 2012, 33, 189–202. 10.1002/jcc.21963. 22042689 PMC3228871

[ref64] Vanommeslaeghe K.; Hatcher E.; Acharya C.; Kundu S.; Zhong S.; Shim J.; Darian E.; Guvench O.; Lopes P.; Vorobyov I.; Mackerell A. D. Jr. CHARMM general force field: A force field for drug-like molecules compatible with the CHARMM all-atom additive biological force fields. J. Comput. Chem. 2010, 31, 671–690. 10.1002/jcc.21367. 19575467 PMC2888302

[ref65] Stone J. An Efficient Library for Parallel Ray Tracing and Animation. M.Sc. Thesis, University of Missouri-Rolla, Rolla, MO, 1998.

[ref66] Humphrey W.; Dalke A.; Schulten K. VMD: Visual molecular dynamics. J. Mol. Graphics 1996, 14, 33–38. 10.1016/0263-7855(96)00018-5. 8744570

[ref67] Marrink S.-J.; Berendsen H. J. C. Simulation of water transport through a lipid membrane. J. Phys. Chem. 1994, 98, 4155–4168. 10.1021/j100066a040.

[ref68] Al-Amoudi A.; Dubochet J.; Norlén L. Nanostructure of the Epidermal Extracellular Space as Observed by Cryo-Electron Microscopy of Vitreous Sections of Human Skin. J. Invest. Dermatol. 2005, 124, 764–777. 10.1111/j.0022-202X.2005.23630.x. 15816835

[ref69] Lindahl V.; Lidmar J.; Hess B. Riemann metric approach to optimal sampling of multidimensional free-energy landscapes. Phys. Rev. E 2018, 98, 023312 10.1103/PhysRevE.98.023312. 30253489

[ref70] Wennberg C. Computational Modeling of Biological Barriers. Ph.D. Thesis, KTH Royal Institute of Technology, Stockholm, Sweden, 2016.

[ref71] Fan S.; Iorga B. I.; Beckstein O. Prediction of octanol-water partition coefficients for the SAMPL6-logP molecules using molecular dynamics simulations with OPLS-AA, AMBER and CHARMM force fields. J. Comput.-Aided Mol. Des 2020, 34, 543–560. 10.1007/s10822-019-00267-z. 31960254 PMC7667952

[ref72] Wang L.; Chen L.; Lian G.; Han L. Determination of partition and binding properties of solutes to stratum corneum. Int. J. Pharm. 2010, 398, 114–122. 10.1016/j.ijpharm.2010.07.035. 20674724

[ref73] Geraniol. Hazardous Substances Data Bank (HSDB). U.S. National Library of Medicine, 2016. https://pubchem.ncbi.nlm.nih.gov/source/hsdb/484 (accessed 2022-10-07).

[ref74] Martins M. A.; Silva L. P.; Ferreira O.; Schröder B.; Coutinho J. A.; Pinho S. A. Terpenes solubility in water and their environmental distribution. J. Mol. Liq. 2017, 241, 996–1002. 10.1016/j.molliq.2017.06.099.

[ref75] (2*E*)-3,7-Dimethyl-2,6-octadien-1-ol. CompTox Chemicals Dashboard. U.S. Environmental Protection Agency, 2016. https://comptox.epa.gov/dashboard/chemical/details/DTXSID8026727 (accessed 2022-10-07).

[ref76] GESTIS-Stoffdatenbank. Institut für Arbeitsschutz der Deutschen Gesetzlichen Unfallversicherung. https://gestis.dguv.de/data?name=491258&lang=en (accessed 2022-10-07).

[ref77] Kasting G. B.; Miller M. A.; Bhatt V. D. A Spreadsheet-Based Method for Estimating the Skin Disposition of Volatile Compounds: Application to N,N.Diethyl-*m*-Toluamide (DEET). J. Occup. Environ. 2008, 5, 633–644. 10.1080/15459620802304245. 18668403

[ref78] Gajjar R. M.; Miller M. A.; Kasting G. B. Evaporation of Volatile Organic Compounds from Human Skin *In Vitro*. Ann. Occup. Hyg. 2013, 57, 853–865. 10.1093/annhyg/met004. 23609116

[ref79] Dias M.; Farinha A.; Faustino E.; Hadgraft J.; Pais J.; Toscano C. Topical delivery of caffeine from some commercial formulations. Int. J. Pharm. 1999, 182, 41–47. 10.1016/S0378-5173(99)00067-8. 10332073

[ref80] Uchida T.; Kadhum W. R.; Kanai S.; Todo H.; Oshizaka T.; Sugibayashi K. Prediction of skin permeation by chemical compounds using the artificial membrane. Strat-MTM. Eur. J. Pharm. Sci. 2015, 67, 113–118. 10.1016/j.ejps.2014.11.002. 25447745

[ref81] Klimovich P. V.; Mobley D. L. Predicting hydration free energies using all-atom molecular dynamics simulations and multiple starting conformations. J. Comput.-Aided Mol. Des. 2010, 24, 307–316. 10.1007/s10822-010-9343-7. 20372973

[ref82] Zhu S. Validation of the Generalized Force Fields GAFF, CGenFF, OPLS-AA and PRODRGFF by Testing Against Experimental Osmotic Coefficient Data for Small Drug-like Molecules. J. Chem. Inf. Model. 2019, 59, 4239–4247. 10.1021/acs.jcim.9b00552. 31557024

[ref83] Singh P.; Roberts M. S. Skin permeability and local tissue concentrations of nonsteroidal anti-inflammatory drugs after topical application. J. Pharmacol. Exp. Ther. 1994, 268, 144–151. 8301551

[ref84] Yue Z.; Li C.; Voth G. A.; Swanson J. M. J. Dynamic Protonation Dramatically Affects the Membrane Permeability of Drug-like Molecules. J. Am. Chem. Soc. 2019, 141, 13421–13433. 10.1021/jacs.9b04387. 31382734 PMC6755907

