# Supplementary material for: Understanding Drug Skin Permeation Enhancers Using Molecular Dynamics Simulations
Source: J Chem Inf Model. 2023 Jul 18;63(15):4900–11. doi: 10.1021/acs.jcim.3c00625 (PMC10428223; doi:10.1021/acs.jcim.3c00625)
Supplement: Supplementary file 1 — ci3c00625_si_001.pdf [file ci3c00625_si_001.pdf]

Supporting Information: Understanding drug skin permeation enhancers using Molecular Dynamics Simulation

Christian Wennberg,[*,†] Magnus Lundborg,[†] Erik Lindahl,[‡,¶] and Lars Norlén[*,§,‖]

†*ERCO Pharma AB, Science for Life Laboratory, 171 65, Solna, Sweden*

‡*Department of Biophysics and Biochemistry, Stockholm University, 106 91, Stockholm, Sweden*

¶*Department of Applied Physics, Swedish e-Science Research Center, KTH Royal Institute of Technology, 106 91, Stockholm, Sweden*

§*Department of Cell and Molecular Biology (CMB), Karolinska Institutet, 171 77, Solna, Sweden*

‖*Dermatology Clinic. Karolinska University Hospital, 171 77, Solna, Sweden*

E-mail: *christian.wennberg@ercopharma.com; *lars.norlen@ki.se

Results

Table S1: Experimental and calculated partition coefficients between water and the skin's barrier structure (w/lip) and octanol and water (ow).

Compound	Log K				Sq. diff.		
	w/lip (exp)	w/lip (calc)[a]	ow (exp)	ow (calc)[b]	w/lip exp-calc	w/lip - ow exp-exp	ow exp-calc
Benzyl Bromide	2.5[c]	3.28	2.92	3.98	0.61	0.18	1.12
Caffeine	0.46[e]	0.31	-0.07	1.89	0.02	0.28	3.83
Cinnamyl alcohol	1.13[c]	0.62	1.95	2.32	0.26	0.67	0.14
Diethylene glycol monobutyl ether	0.48[c]	-0.47	0.56	1.48	0.90	0.01	0.84
Estradiol	2.25[d]	2.65	3.86	5.38	0.16	2.59	2.31
Eugenol	1.67[c]	1.74	2.27	3.10	0.01	0.36	0.68
Geraniol	1.89[c]	2.06	3.56	3.99	0.03	2.79	0.18
Ibuprofen	2.00[c]	2.08	3.97	4.43	0.01	3.88	0.20
Iso-eugenol	2.05[c]	2.17	3.04	3.39	0.01	0.98	0.12
Lidocaine	1.38[d]	1.68	2.32	4.28	0.09	0.88	3.83
Methylparaben	0.85[c]	-0.43	1.96	1.71	1.64	1.23	0.06
Naphthalene	3.42[c]	2.86	3.30	3.58	0.31	0.01	0.08
Progesterone	3.03[d]	3.6	3.74	5.41	0.32	0.50	2.79
Testosterone	1.92[f]	2.17	3.32	4.93	0.06	1.95	2.61
Triclosan	4.34[c]	4.42	4.76	5.75	0.01	0.18	0.98
Vanillin	1.01[c]	0.08	1.21	1.41	0.86	0.04	0.04
				**avg. Sq. diff. :**	0.33	1.03	1.24
				**RMSE :**	0.58	1.02	1.11

[a] Calculated as $\frac{\Delta G_{w-lip}}{RT \times ln(10)}$
[b] Calculated as $\frac{\Delta G_{ow}}{RT \times ln(10)}$
[c] Ref. S1
[d] Ref. S2
[e] Average of values from Ref. S1 and S3
[f] Average of values from Ref. S1 and S2

Table S2: Number of additional molecules inserted into the skin's barrier structure system, for each formulation modeled in this work.

Formulation	Molecules to insert[a]	Inserted molecules[b]	Mass-% increase[c]
Water	100	100	1.3
Water/Geraniol (100 mg/L)	100 / 12	100 / 12	2.7
Water/Geraniol (200 mg/L)	100 / 24	100 / 24	4.1
Water/Geraniol (686 mg/L)	100 / 84	100 / 84	11.0
Water/Stearic acid	100	100	1.3
Water/Lauric acid (16 inserted)	100 / 16	100 / 16	3.7
Water/Lauric acid (24 inserted)	100 / 24	100 / 24	4.9
Water/Lauric acid (36 inserted)	100 / 36	100 / 36	6.7
Water/Lauric acid (54 inserted)	100 / 54	100 / 54	9.4
Water/Thymol (100 mg/L)	100 / 104	100 / 104	12.9
Water/Thymol (200 mg/L)	100 / 208	100 / 208	24.5
Water/Thymol (900 mg/L)	100 / 910	100 / 910	102.8
Water/Ethanol	81 / 46	42 / 36	1.8
Water/Ethanol/Eucalyptol (26 inserted)	82 / 43 / 26	48 / 34 / 26	4.8
Water/Ethanol/Eucalyptol (52 inserted)	82 / 43 / 52	48 / 34 / 52	7.7
Water/Ethanol/Oleic Acid (18 inserted)	80 / 45 / 18	44 / 36 / 18	5.6
Water/Ethanol/Oleic Acid (36 inserted)	80 / 45 / 36	44 / 36 / 36	9.4
Water/Ethanol/Oleic Acid (54 inserted)	80 / 45 / 54	44 / 36 / 54	13.1

[a] According to eq. 3
[b] After limiting the increase in mass in the headgroup region to a maximum of 2.5 times the mass of the water molecules in the headgroups.
[c] Compared to total mass of pure skin's barrier structure

Table S3: All calculated metronidazole permeability coefficients and the corresponding enhancement ratios (ER) for different permeation enhancers (PEs).

Permeation enhancer	***In vitro***[a]		**Calculated**	
	**$\log K_P$** (cm/h)	**ER**	**$\log K_P$** (cm/h)	**ER**
Water	−2.89±0.03	1	−3.79±0.24	1
Lauric acid (16 inserted)	−1.78±0.07	13	−3.73±0.23	1
Lauric acid (24 inserted)			−2.97±0.30	7
Lauric acid (36 inserted)			−3.02±0.19	6
Lauric acid (54 inserted)			−2.86±0.22	9
Geraniol (100 mg/L)	−2.17±0.04	5	−3.66±0.23	1.4
Geraniol (200 mg/L)			−2.74±0.21	11.3
Geraniol (686 mg/L)			−2.13±0.16	46
Stearic acid	−2.80±0.03	1	−3.79±0.24	1
Thymol (100 mg/L)	> −1	> 100	−2.59±0.21	16
Thymol (200 mg/L)			−1.39±0.26	256
Thymol (900 mg/L)			0.91±0.12	5000

[a] Experimental data calculated from Table 3 in ref. S4

Table S4: Effect of modifying the calculated permeability values of naproxen based on the pH-pK_a difference according to eq. 6

	In vitro[a]		**Calculated (modified)**		**Calculated (no modification)**	
	**$\log K_P$** (cm/h)	**ER**	**$\log K_P$** (cm/h)	**ER**	**$\log K_P$** (cm/h)	**ER**
**Naproxen permeability**						
Water/Ethanol[b]	−3.34±0.07	1.0	−2.85±0.10	1.0	−2.15±0.09	1.0
Oleic acid (18 inserted)[c]	−1.73±0.02	41	−2.39±0.13	2.9	−2.31±0.13	0.7
Oleic acid (36 inserted)[c]			−1.68±0.11	14.8	−1.60±0.21	3.5
Oleic acid (54 inserted)[c]			−1.76±0.20	12.5	−1.69±0.17	3.0
Eucalyptol (26 inserted)[d]	−2.44±0.04	7.9	−2.46±0.11	2.5	−2.43±0.11	0.5
Eucalyptol (52 inserted)[d]			−2.02±0.17	2.5	−1.99±0.17	1.4

[a] Experimental data calculated from J_{ss} values in Table 1 in ref. S5
[b] 40% water, 60% ethanol
[c] 40% water, 60% ethanol, 3% w/v Oleic acid
[d] 40% water, 60% ethanol, 5% w/v Eucalyptol

Table S5: Composition of simulated formulations. Geraniol, stearic acid, lauric acid and thymol have such low solubilities that they were assumed to have negligible effect on the solubility of metronidazole and thus their simulation boxes were identical to water.

**Formulation system**	**No. molecules**	**Simulation box size X/Y/Z (nm)**	**No. molecules/nm^3**
Water	2664	4.01 / 4.01 / 5.02	33
Octanol/water (used to calculate $LogK_{octanol-water}$)	288 / 108	4.3 / 4.3 / 4.3	3.6 / 1.4
Water/Geraniol (100 mg/L) [a]	2664 / 0	4.01 / 4.01 / 5.02	33 / 4.0*e*-4
Water/Geraniol (200 mg/L) [b]	2664 / 0	4.01 / 4.01 / 5.02	33 / 8.0*e*-4
Water/Geraniol (686 mg/L)[c]	2664 / 0	4.01 / 4.01 / 5.02	33 / 2.7*e*-3
Water/Stearic acid[d]	2664 / 0	4.01 / 4.01 / 5.02	33 / 7.2*e*-9
Water/Lauric acid (all systems)[e]	2664 / 0	4.01 / 4.01 / 5.02	33 / 2.0*e*-7
Water/Thymol (100 mg/L) [f]	2664 / 0	4.01 / 4.01 / 5.02	33 / 4.0*e*-4
Water/Thymol (200 mg/L) [g]	2664 / 0	4.01 / 4.01 / 5.02	33 / 8.0*e*-4
Water/Thymol (900 mg/L)[h]	2664 / 0	4.01 / 4.01 / 5.02	33 / 2.8*e*-3
Water/Ethanol[i]	1155 / 338	4.08 / 4.08 / 4.08	17 / 4.98
Water/Ethanol/Eucalyptol (all systems)[j]	1155 / 338 / 20	4.20 / 4.20 / 4.20	15.59 / 4.56 / 0.27
Water/Ethanol/Oleic Acid (all systems)[k]	1155 / 338 / 6	4.15 / 4.15 / 4.15	16.16 / 4.73 / 0.08

[a] Simulation box would contain 4.0*e*-4 molecules / nm^3
[b] Simulation box would contain 8.0*e*-4 molecules / nm^3
[c] Simulation box would contain 2.7*e*-3 molecules / nm^3
[d] Saturated solution contains 3.4*e*-6 g/L, simulation box would contain 7.2*e*-9 molecules / nm^3
[e] Saturated solution contains 6.5*e*-5 g/L, simulation box would contain 2.0*e*-7 molecules / nm^3
[f] Simulation box would contain 4*e*-4 molecules / nm^3
[g] Simulation box would contain 8*e*-4 molecules / nm^3
[h] Simulation box would contain 2.8*e*-3 molecules / nm^3
[i] 40% water, 60% ethanol, 50% ethanol evaporation
[j] 40% water, 60% ethanol, 5% w/v Eucalyptol, 50% ethanol evaporation
[k] 40% water, 60% ethanol, 3% w/v Oleic acid, 50% ethanol evaporation

Table S6: Permeability coefficients and enhancement ratios (ERs) calculated for caffeine and naproxen.

	In vitro[a]		Calculated	
	**logK$_P$** (cm/h)	**ER**	**logK$_P$** (cm/h)	**ER**
**Caffeine permeability**				
Water	−4.15±0.04	1.0	−3.0±0.20	1
Water/Ethanol[b]	−3.50±0.02	4.5	−2.28±0.24	5
Oleic acid (18 inserted)[c]	−1.91±0.02	175	−1.92±0.18	12
Oleic acid (36 inserted)[c]			−2.21±0.20	6
Oleic acid (54 inserted)[c]			−2.28±0.20	5
Eucalyptol (26 inserted)[d]	−1.99±0.01	148	−2.64±0.23	2
Eucalyptol (52 inserted)[d]			−2.00±0.25	10
**Naproxen permeability**				
Water/Ethanol[b]	−3.34±0.07	1.0	−2.85±0.10	1.0
Oleic acid (18 inserted)[c]	−1.73±0.02	41	−2.39±0.13	2.9
Oleic acid (36 inserted)[c]			−1.68±0.11	14.8
Oleic acid (54 inserted)[c]			−1.76±0.20	12.5
Eucalyptol (26 inserted)[d]	−2.44±0.04	7.9	−2.46±0.11	2.5
Eucalyptol (52 inserted)[d]			−2.02±0.17	6.8

[a] Experimental data from Table 1 in ref. S5
[b] 40% water, 60% ethanol
[c] 40% water, 60% ethanol, 3% w/v Oleic acid
[d] 40% water, 60% ethanol, 5% w/v Eucalyptol

Table S7: Calculated number of internal h-bonds between all lipids in each skin system with inserted permeation enhancers.

**Skin system**	**Average number of lipid H-bonds**	**Normalized**
Pure skin system	287	1
Geraniol (100 mg/L)	246	0.86
Geraniol (200 mg/L)	238	0.83
Geraniol (686 mg/L)	232	0.81
Lauric acid (16 inserted)	238	0.83
Lauric acid (24 inserted)	238	0.83
Lauric acid (36 inserted)	230	0.80
Lauric acid (54 inserted)	222	0.77
Thymol (100 mg/L)	246	0.86
Thymol (200 mg/L)	218	0.76
Thymol (900 mg/L)	205	0.71
Water/Ethanol	249	0.87
Oleic acid (18 inserted)	252	0.88
Oleic acid (36 inserted)	250	0.87
Oleic acid (54 inserted)	244	0.85
Eucalyptol (26 inserted)	245	0.85
Eucalyptol (52 inserted)	237	0.83

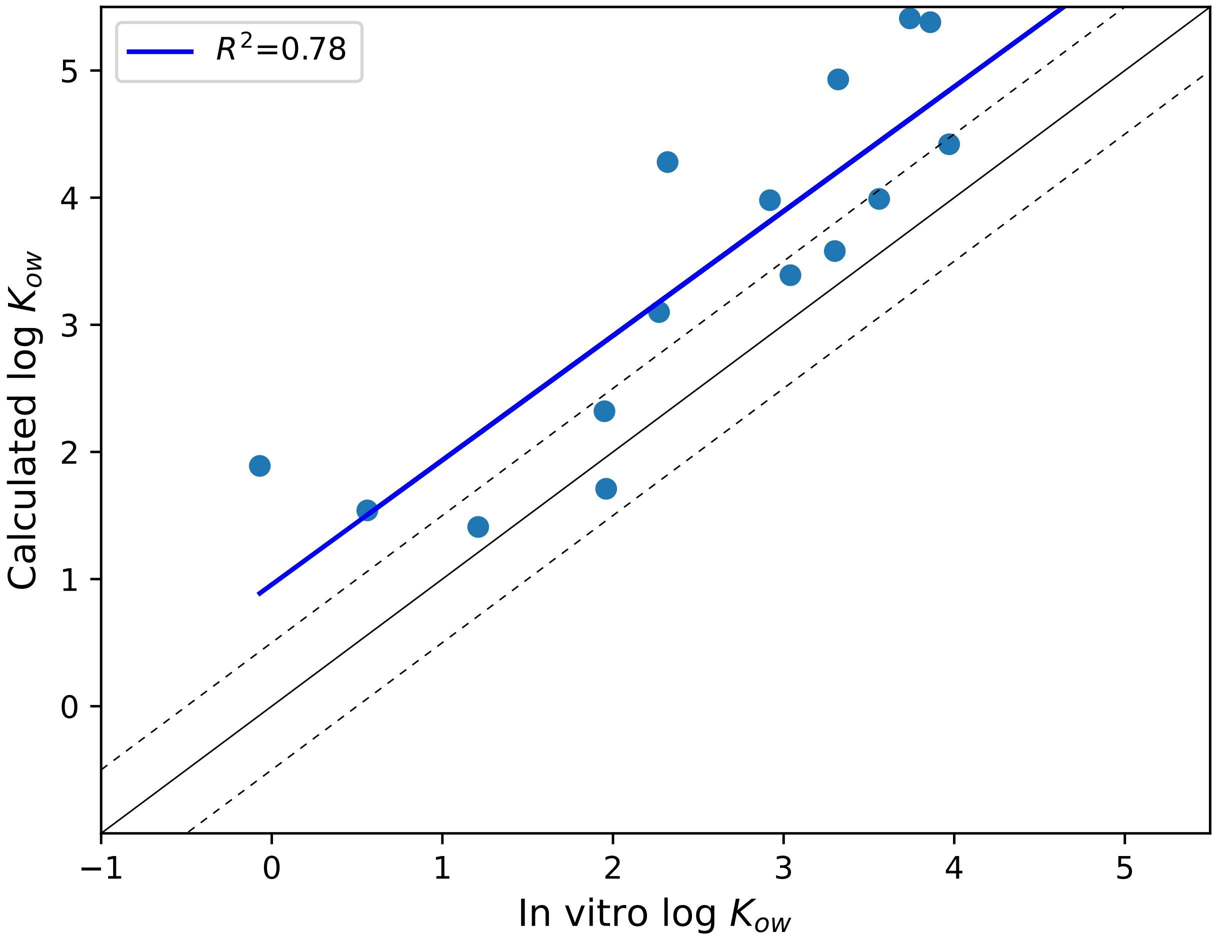

Figure S1: Correlation between calculated and *in vitro* octanol-water partitioning. Dashed lines above and below the identity line corresponds to ±0.5 log-units. Data also available in Table S1.

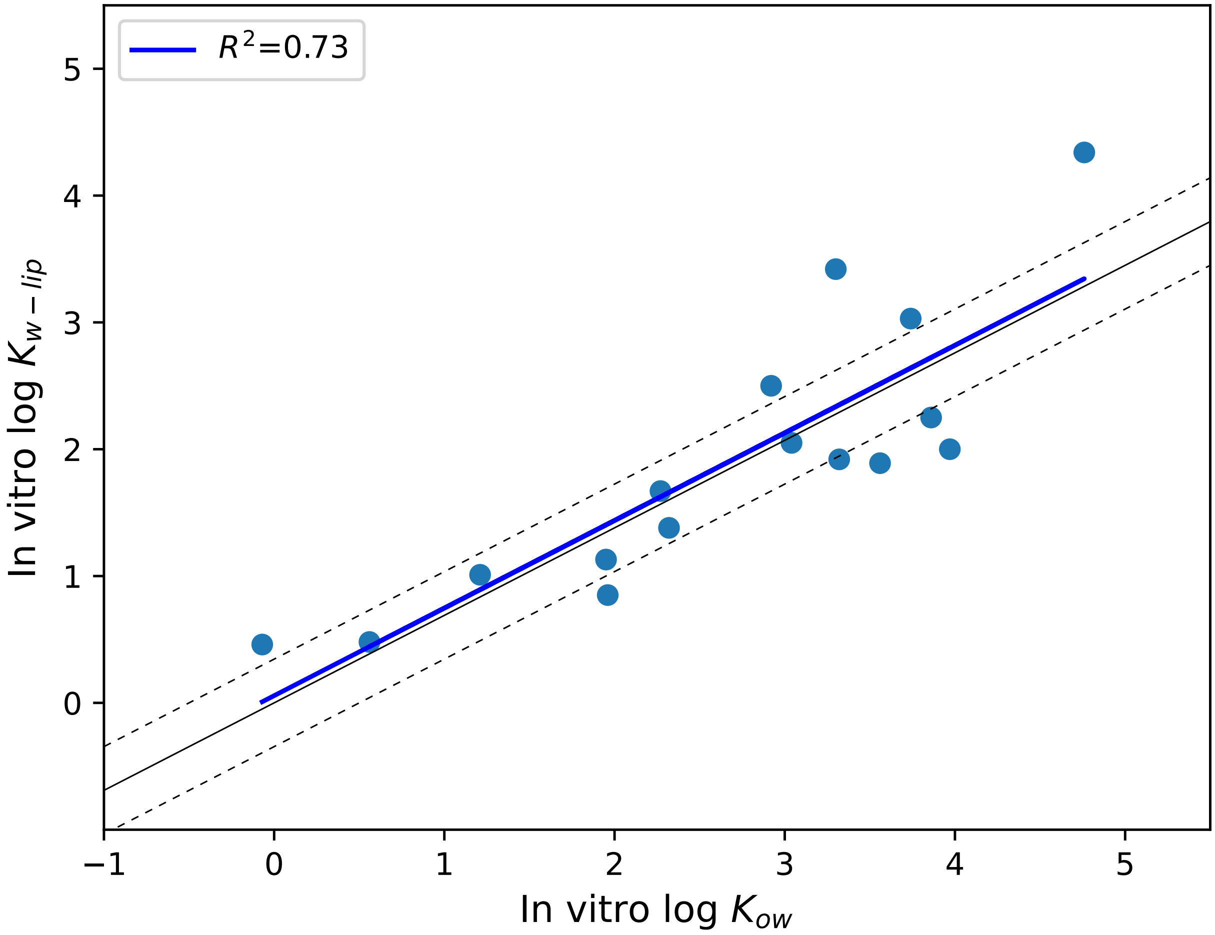

Figure S2: Correlation between *in vitro* partition coefficients in octanol-water and from water into the skin's barrier structure. Plotted in black: $y = 0.69 log K_{ow}$. Dashed lines above and below the black line corresponds to ±0.5 log-units. Data also available in Table S1. Experimental data from Ref. S1–S3

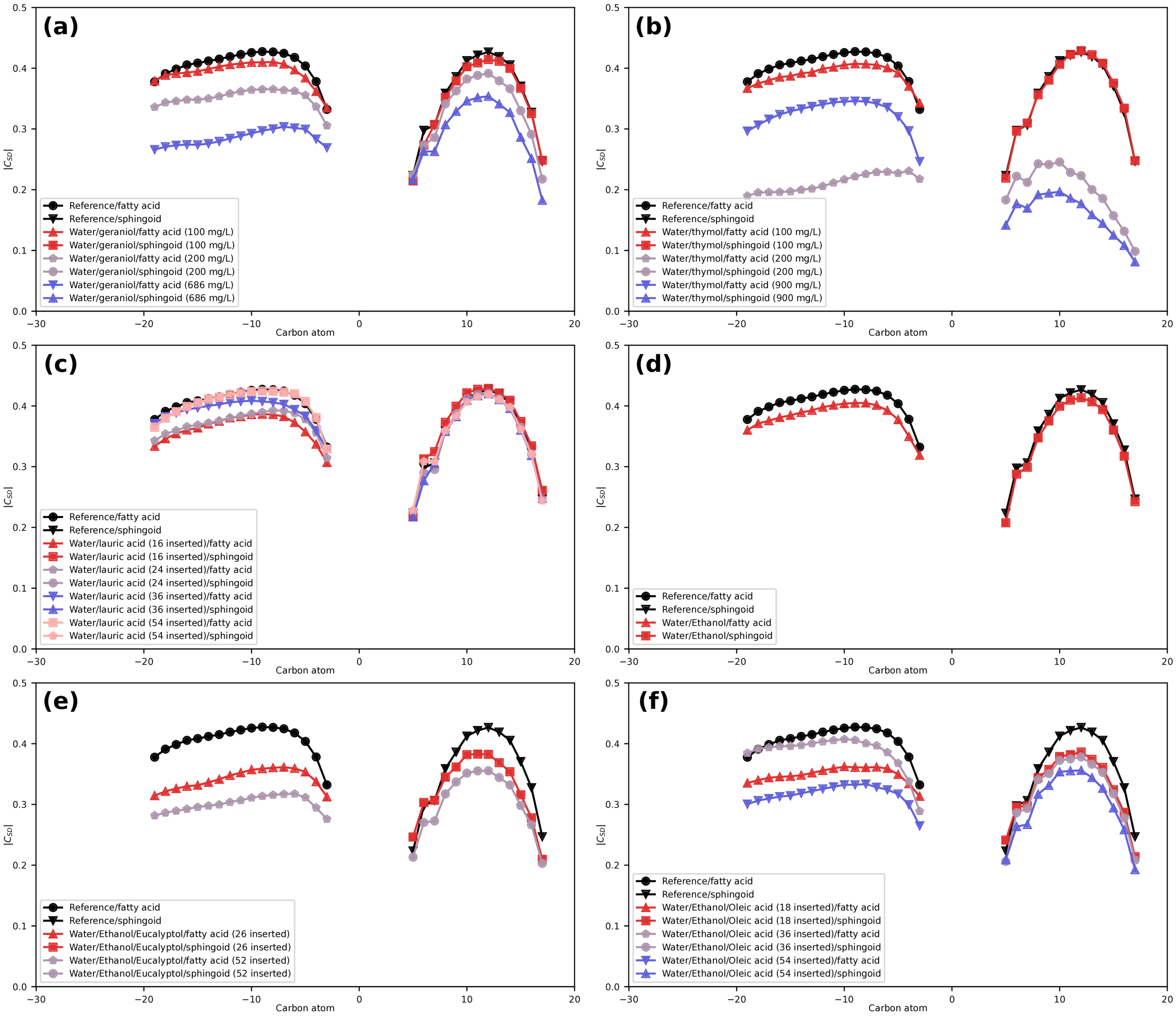

Figure S3: Calculated deuterium order parameters for the ceramide molecules in the different skin barrier structure systems with inserted permeation enhancers (PEs). **(a):** Water/geraniol, **(b):** Water/thymol, **(c):** Water/lauric acid, **(d):** Water/Ethanol, **(e):** Water/ethanol/eucalyptol, and **(f):** Water/ethanol/oleic acid. The fatty acid chain of the ceramides are represented with negative carbon atom indices while the sphingoid chain have positive indices. The head group of the ceramides are positioned at carbon atom index 0. In each plot (a-f) the order parameters for the reference skin's barrrier structure system, without any additional excipients, are shown in black.

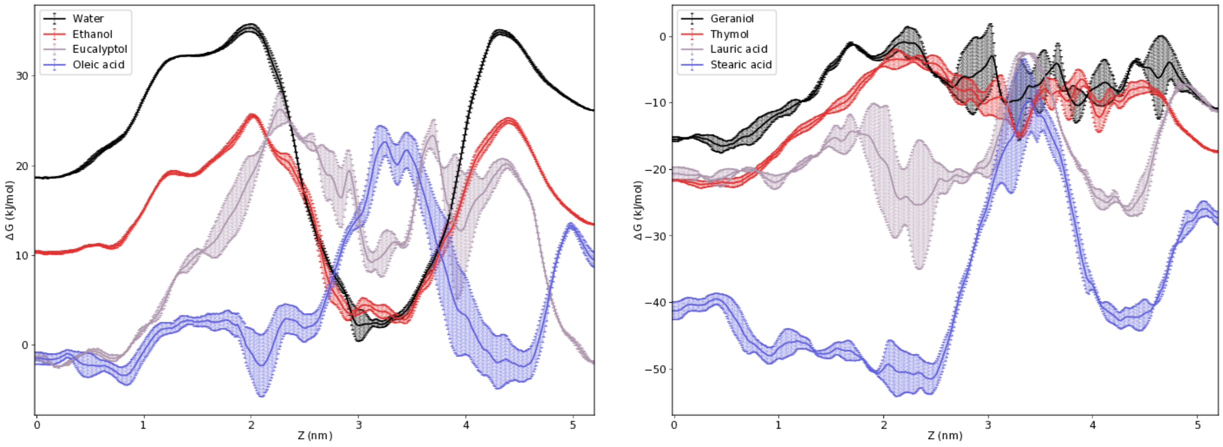

Figure S4: Potentials of mean force across the skin barrier structure for each permeability enhancer (PE). The x-axis corresponds to one half of the skin lipid bilayer, with the center of the bilayer (between the fatty acid chains of the ceramides) situated at $z = 0$ nm, the lipid headgroups situated at $z \sim 3.2$ nm.

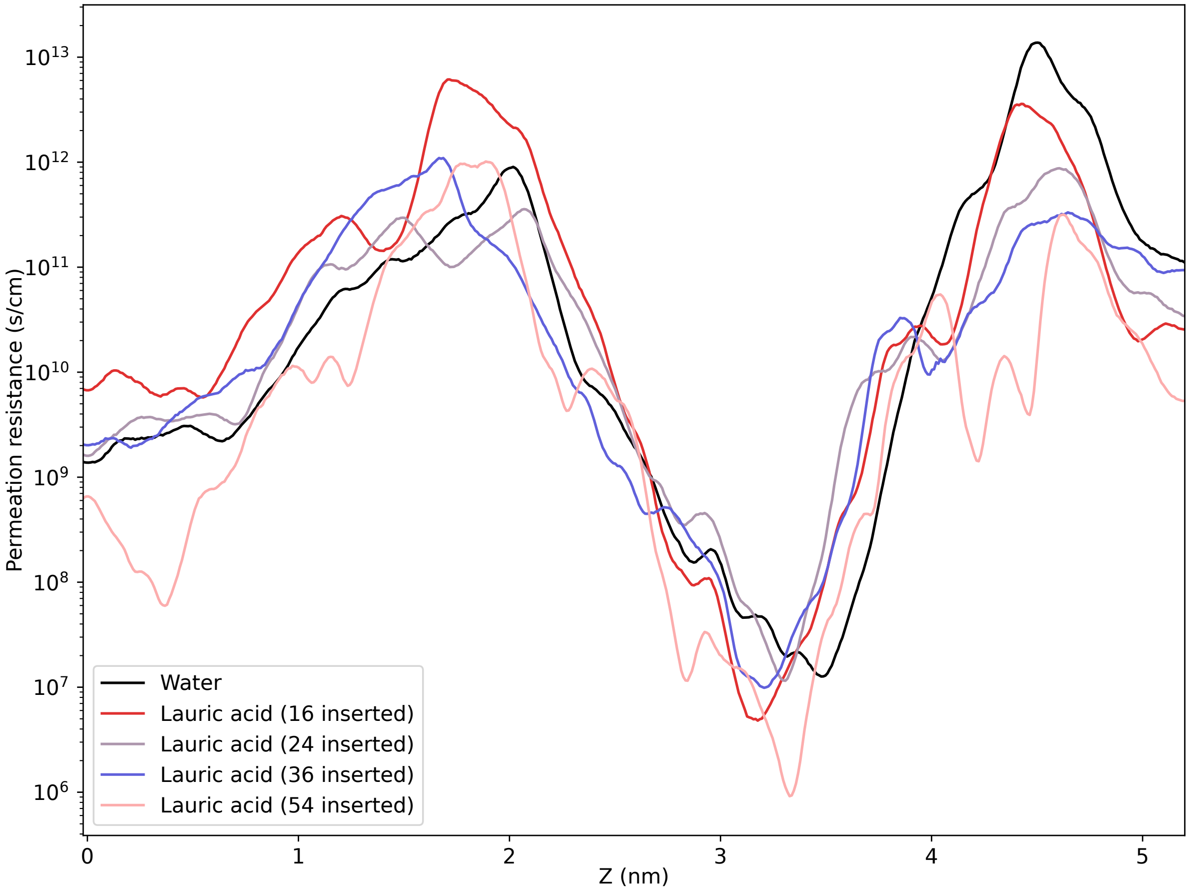

Figure S5: Calculated permeation resistance profiles for metronidazole across the skin barrier structure with lauric acid as a permeation enhancer (PE). The x-axis corresponds to one half of the skin lipid bilayer, with the center of the bilayer (between the fatty acid chains of the ceramides) situated at $z = 0$ nm, the lipid headgroups situated at $z \sim 3.2$ nm.

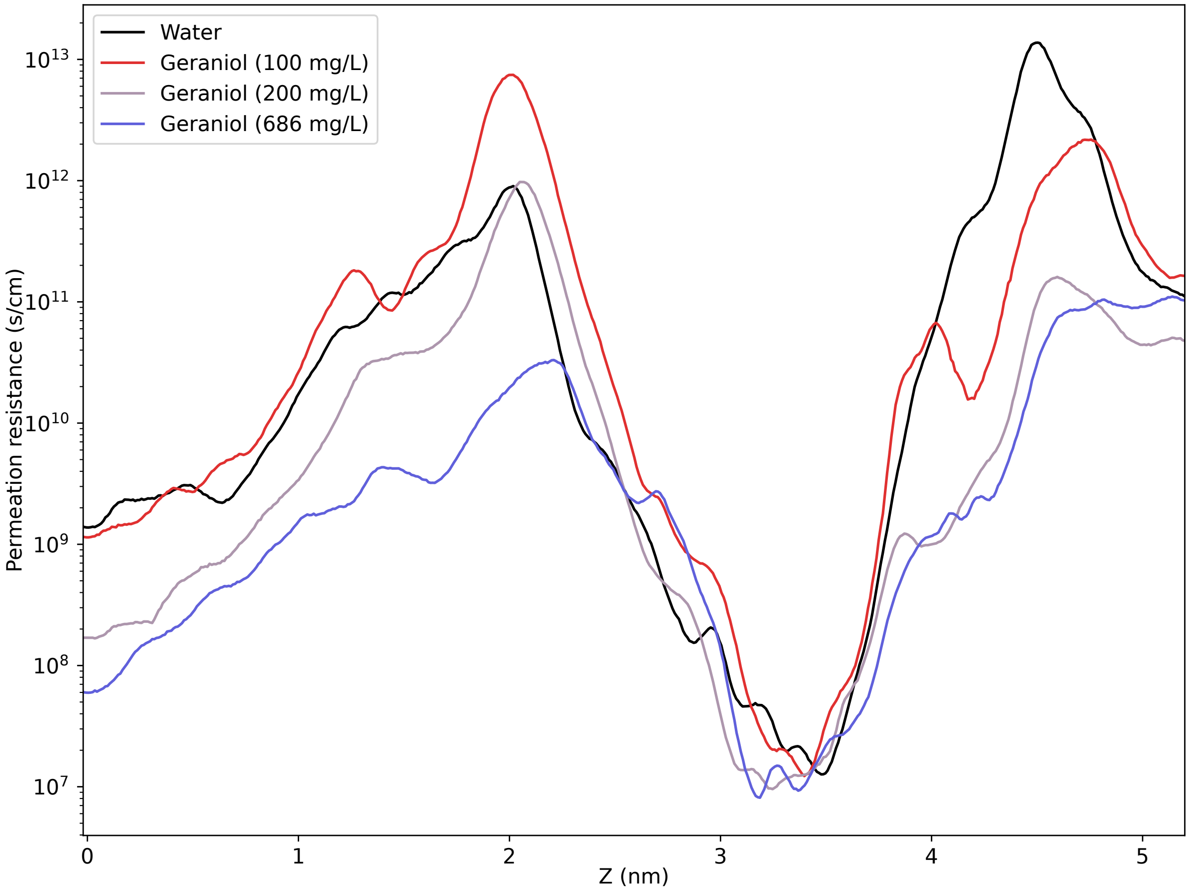

Figure S6: Calculated permeation resistance profiles for metronidazole across the skin barrier structure with geraniol as a permeation enhancer (PE). The x-axis corresponds to one half of the skin lipid bilayer, with the center of the bilayer (between the fatty acid chains of the ceramides) situated at $z = 0$ nm, the lipid headgroups situated at $z \sim 3.2$ nm.

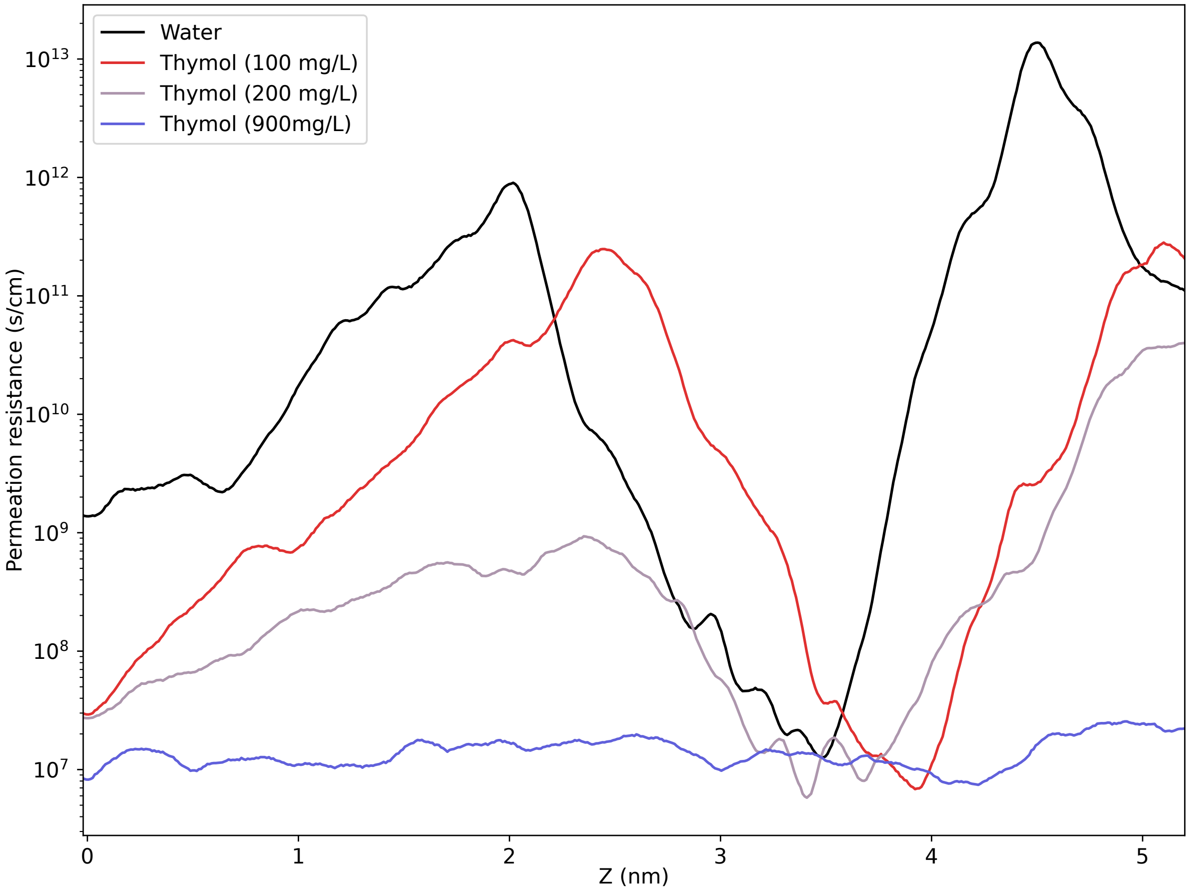

Figure S7: Calculated permeation resistance profiles for metronidazole across the skin barrier structure with thymol as a permeation enhancer (PE). The x-axis corresponds to one half of the skin lipid bilayer, with the center of the bilayer (between the fatty acid chains of the ceramides) situated at $z = 0$ nm, the lipid headgroups situated at $z \sim 3.2$ nm.

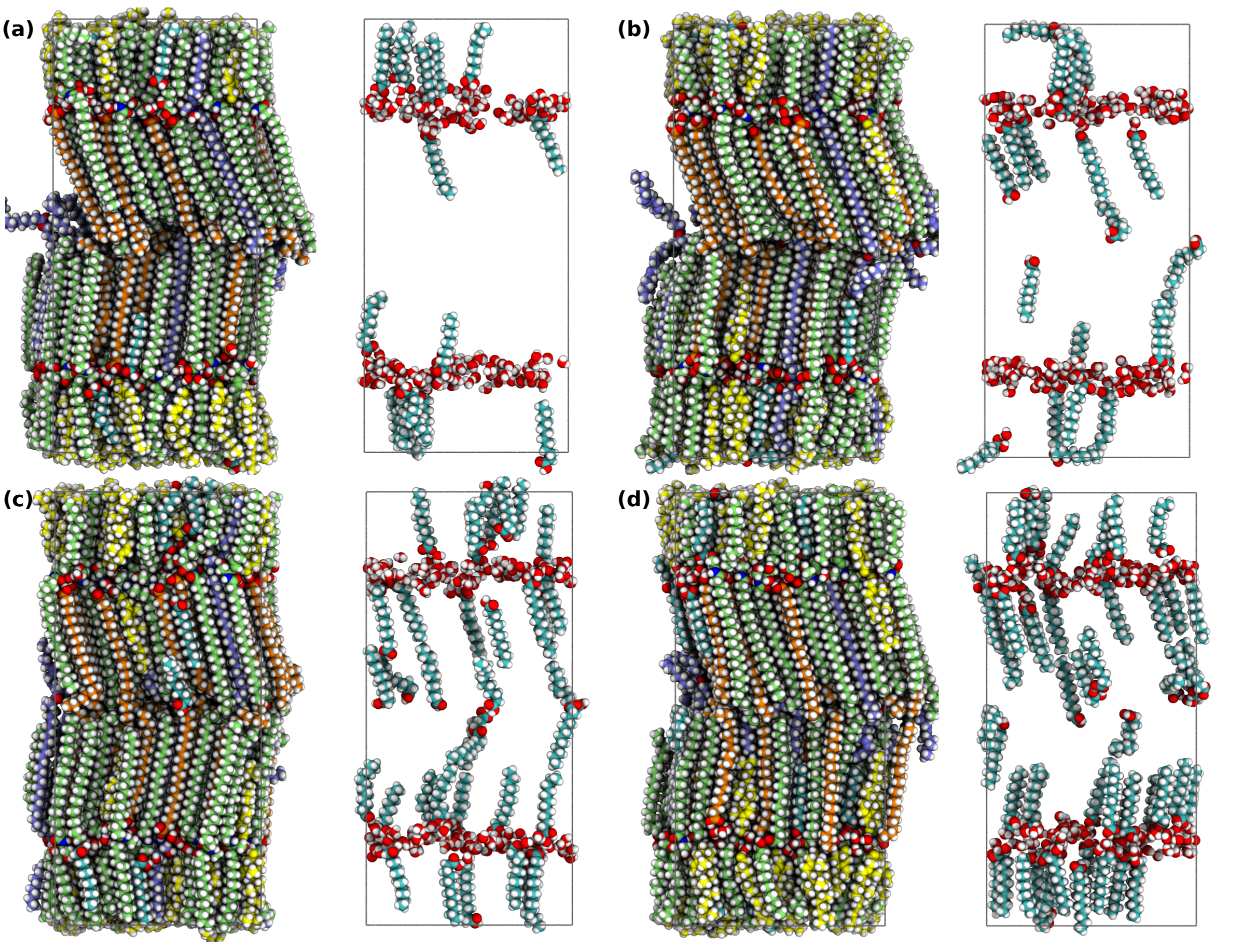

Figure S8: The skin barrier structure systems with inserted lauric acid. **(a)**: 16 molecules inserted, **(b)**: 24 molecules inserted, **(c)**: 36 molecules inserted, **(d)**: 54 molecules inserted.

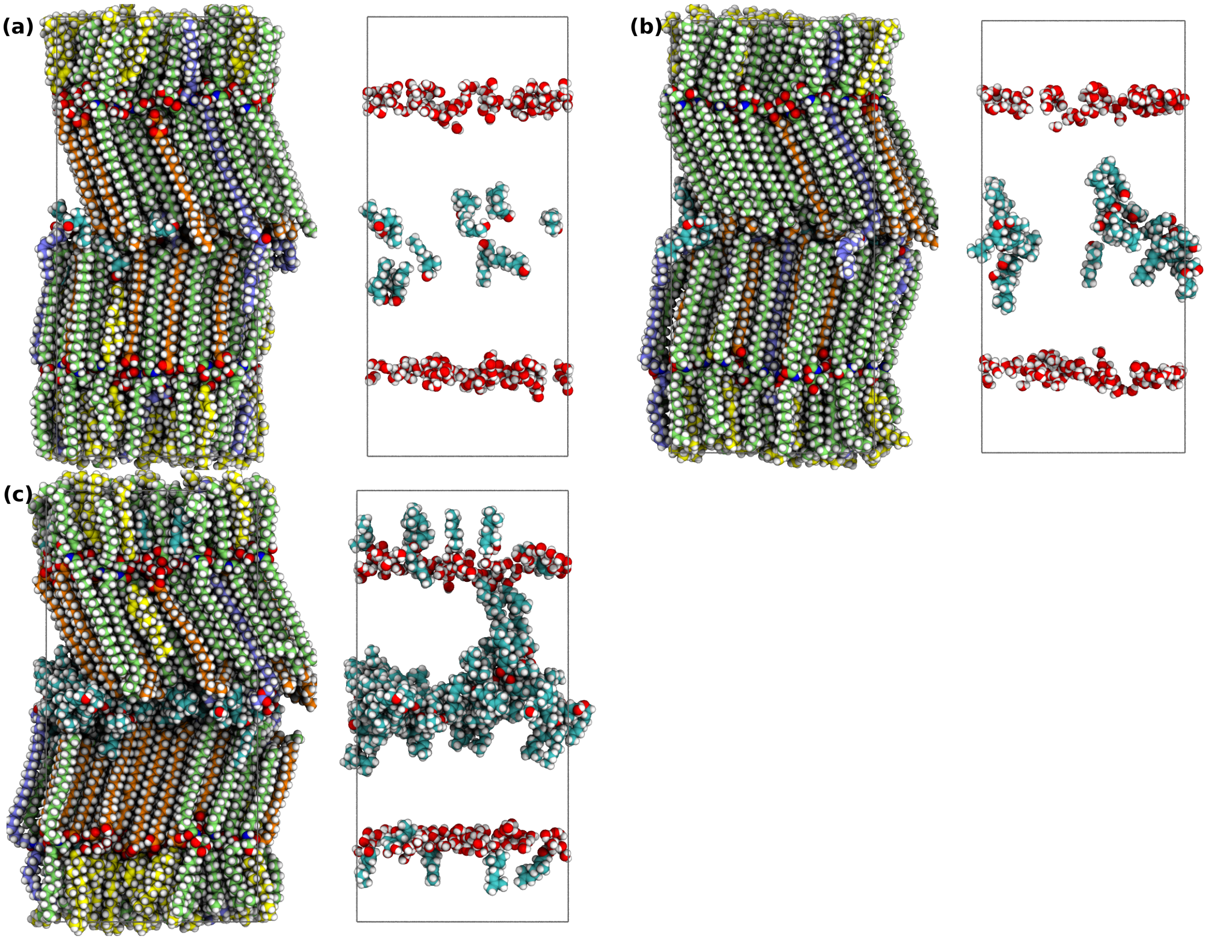

Figure S9: The skin barrier structure systems with inserted geraniol from the three different formulations simulated. **(a)**: 100 mg/L geraniol in formulation, **(b)**: 200 mg/L geraniol in formulation, **(c)**: 686 mg/L geraniol in formulation.

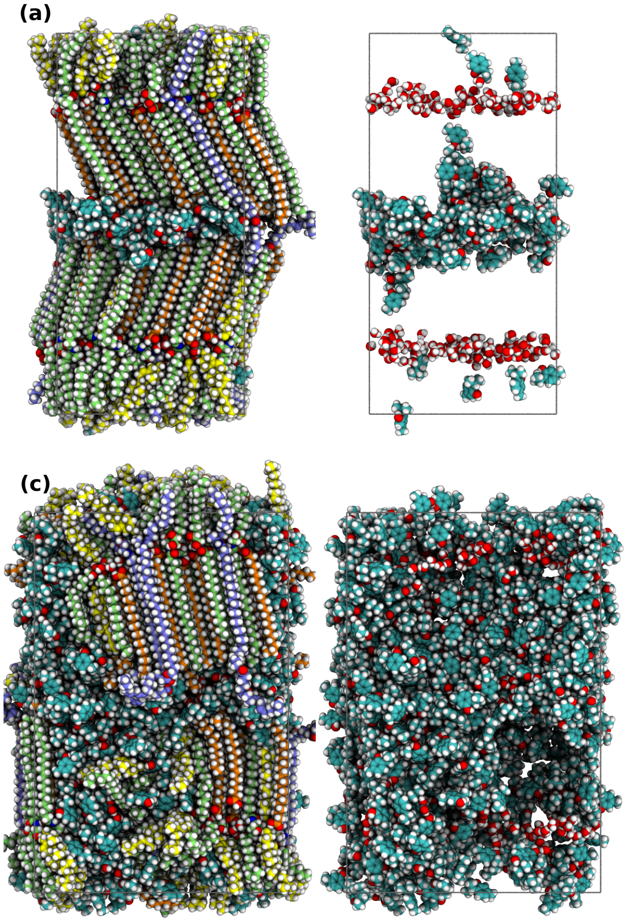

Figure S10: The skin barrier structure systems with inserted thymol from the three different formulations simulated. **(a)**: 100 mg/L thymol in formulation, **(b)**: 200 mg/L thymol in formulation, **(c)**: 900 mg/L thymol in formulation.

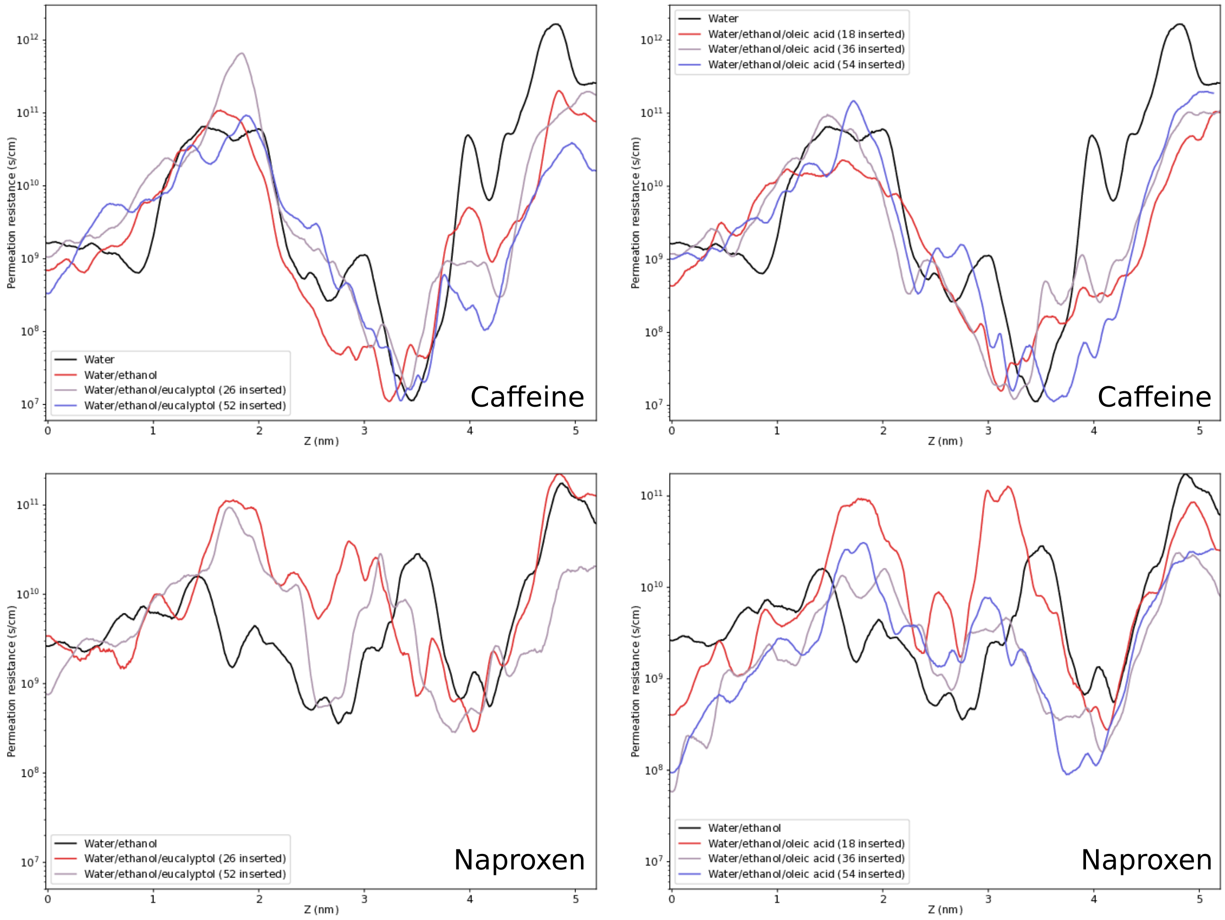

Figure S11: Calculated permeation resistance profiles for caffeine and naproxen across the skin barrier structure. The x-axis corresponds to one half of the skin lipid bilayer, with the center of the bilayer (between the fatty acid chains of the ceramides) situated at $z = 0$ nm, the lipid headgroups situated at $z \sim 3.2$ nm.

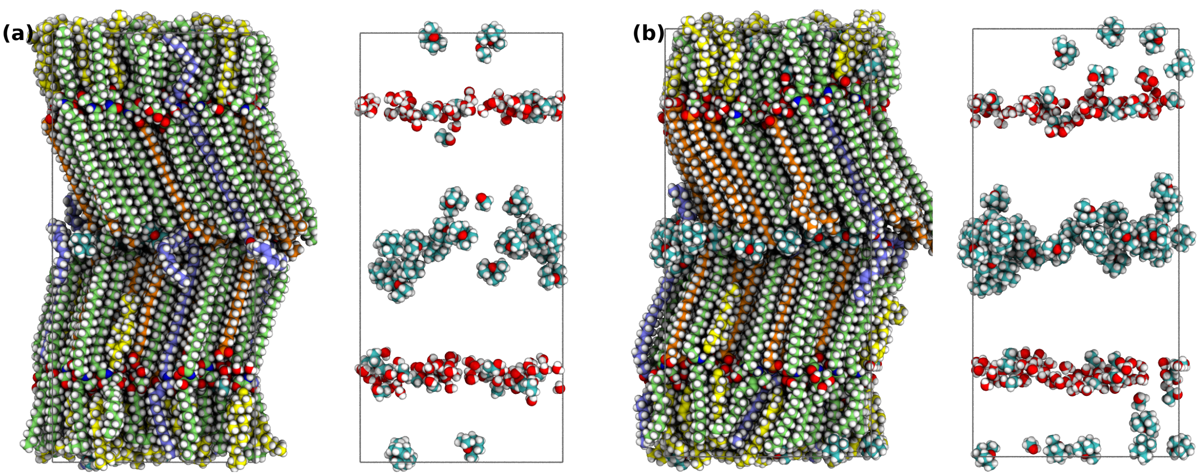

Figure S12: The skin barrier structure systems with inserted eucalyptol. **(a)**: 26 molecules inserted, **(b)**: 52 molecules inserted.

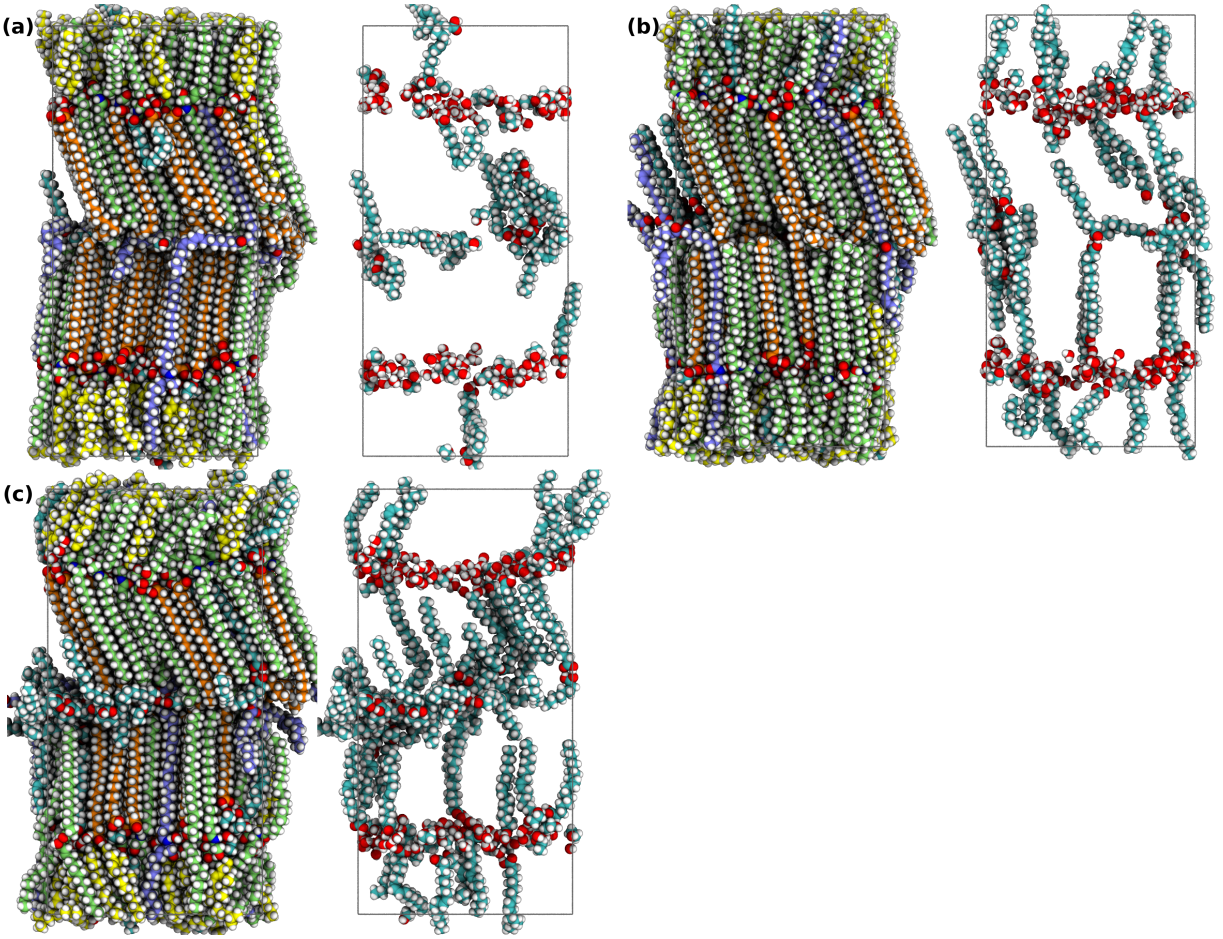

Figure S13: The skin barrier structure systems with inserted oleic acid. **(a)**: 18 molecules inserted, **(b)**: 36 molecules inserted, **(c)**: 54 molecules inserted.

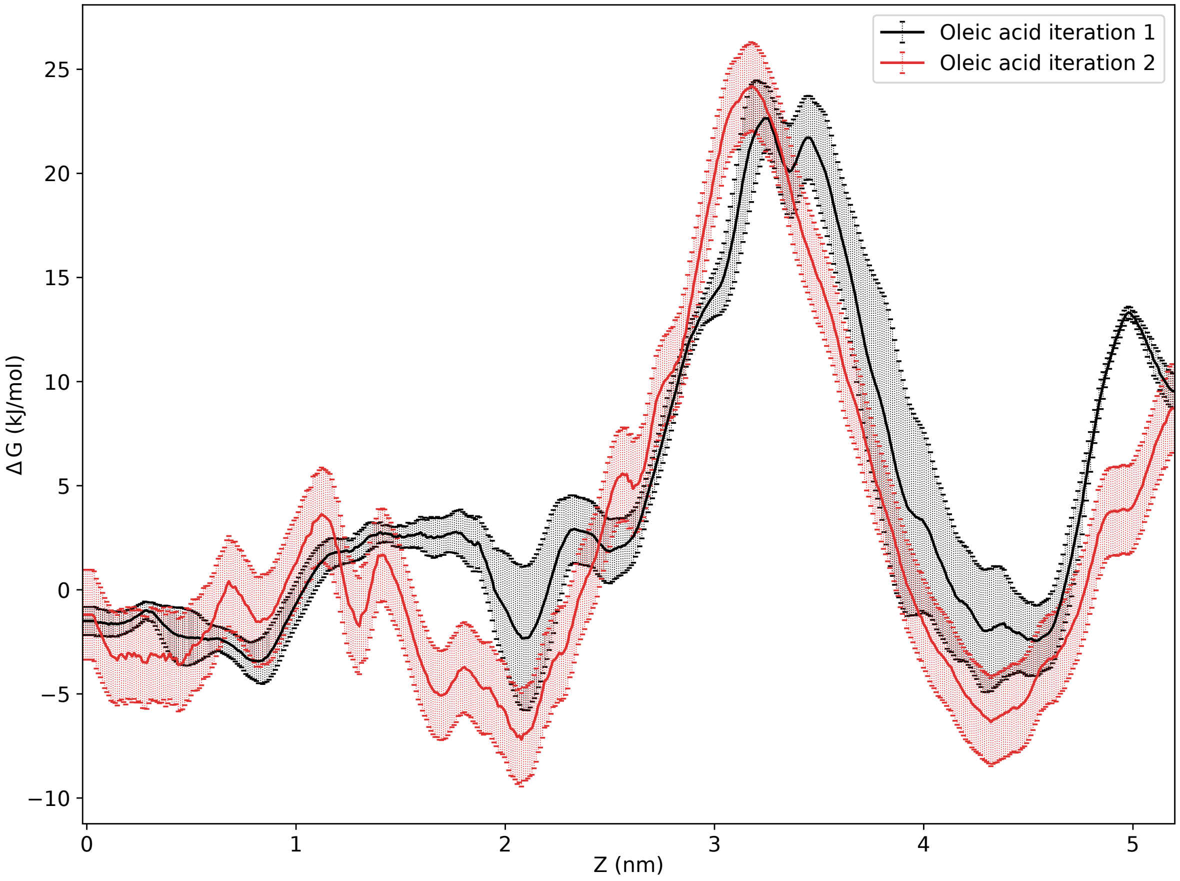

Figure S14: Calculated potentials of mean force (PMF) for oleic acid across the skin barrier structure system. Iteration 1: PMF of oleic acid across the pure skin lipid bilayer. Iteration 2: PMF of oleic acid across the skin lipid bilayer with oleic acid added to the system based on the PMF from iteration 1 (same system as in Fig. S13-**(a)**).

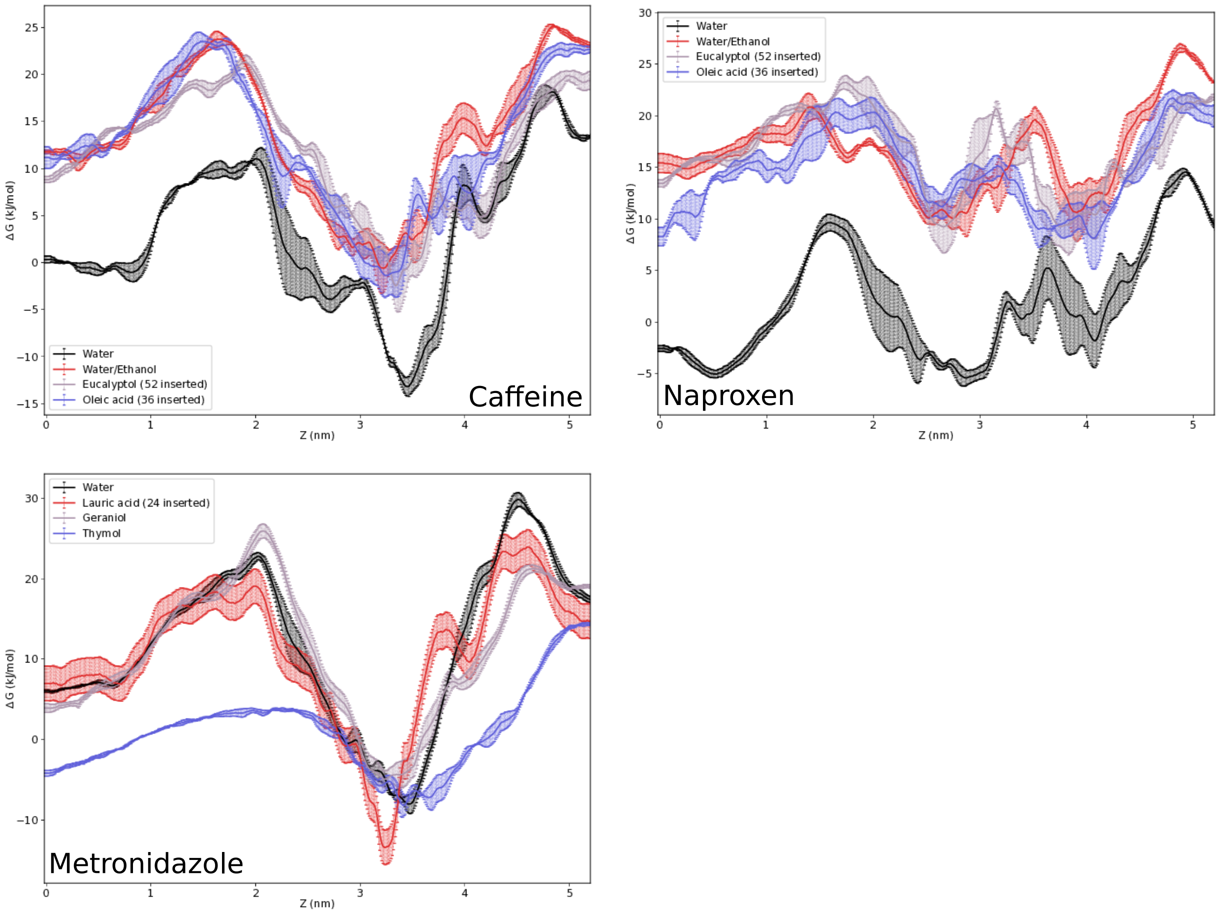

Figure S15: Potentials of mean force for caffeine, naproxen and metronidazole through some of the different skin barrier structure systems in Table S2, calibrated against the corresponding formulations in Table S5.

References

(S1) Ellison, C. A.; Tankersley, K. O.; Obringer, C. M.; Carr, G. J.; Manwaring, J.; Rothe, H.; Duplan, H.; Géniès, C.; Grégoire, S.; Hewitt, N. J.; Jamin, C. J.; Klaric, M.; Lange, D.; Rolaki, A.; Schepky, A. Partition coefficient and diffusion coefficient determinations of 50 compounds in human intact skin, isolated skin layers and isolated stratum corneum lipids. *Toxicol In Vitro* **2020**, *69*, 104990.

(S2) Johnson, M. E.; Blankschtein, D.; Langer, R. Evaluation of solute permeation through the stratum corneum: lateral bilayer diffusion as the primary transport mechanism. *J. Pharm. Sci.* **1997**, *86*, 1162–1172.

(S3) Hansen, S.; Henning, A.; Naegel, A.; Heisig, M.; Wittum, G.; Neumann, D.; Kostka, K.-H.; Zbytovska, J.; Lehr, C.-M.; Schaefer, U. F. In-silico model of skin penetration based on experimentally determined input parameters. Part I: experimental determination of partition and diffusion coefficients. *Eur. J. Pharm. Biopharm.* **2008**, *68*, 352–367.

(S4) Pham, Q. D.; Björklund, S.; Engblom, J.; Topgaard, D.; Sparr, E. Chemical penetration enhancers in stratum corneum — Relation between molecular effects and barrier function. *J. Control. Release* **2016**, *232*, 175–187.

(S5) Abd, E.; Benson, H. A.; Mohammed, Y. H.; Roberts, M. S.; Grice, J. E. Permeation Mechanism of Caffeine and Naproxen htrough in vitro Human Epidermis: Effect of Vehicles and Penetration Enhancers. *Skin Pharmacol. Physiol.* **2019**, *32*, 132–141.
